# Ordered assembly of the *Vibrio cholerae* biofilm exopolysaccharide defined by lipid-linked intermediate profiling

**DOI:** 10.1128/mbio.01096-26

**Published:** 2026-07-14

**Authors:** Manoj K. Dooda, Anna Potapova, Sofia Piepoli, Evgeny Vinogradov, Fitnat H. Yildiz, Jerry M. Troutman

**Affiliations:** 1 Department of Biological Sciences, University of North Carolina at Charlotte 14727, Charlotte, North Carolina, USA; 2 Department of Microbiology and Environmental Toxicology, University of California Santa Cruz 8787, Santa Cruz, California, USA; 3 National Research Council 6356 , Ottawa, Ontario, Canada; 4 Department of Chemistry, University of North Carolina at Charlotte 14727 , Charlotte, North Carolina, USA; Michigan State University, East Lansing, Michigan, USA

**Keywords:** *Vibrio* polysaccharide, *Vibrio cholerae*, glycan, glycosyltransferase, biofilms, exopolysaccharide

## Abstract

Biofilm formation underlies the environmental persistence and transmission of *Vibrio cholerae*, the etiological agent of cholera. The Vibrio polysaccharide (VPS) is the principal structural component of the mature biofilm matrix, yet the enzymatic logic governing its assembly has remained incompletely defined. VPS is synthesized as two closely related polymers that share a tetrasaccharide repeat unit but differ at a single monosaccharide position. Here, we systematically define VPS assembly by integrating targeted gene deletions with liquid chromatography-mass spectrometry profiling of lipid-linked intermediates, and comparative structural modeling of biosynthetic enzymes. Our results establish that VPS is produced through an ordered Wzx/Wzy-dependent pathway. VpsL functions as the initiating phosphoglycosyltransferase, generating bactoprenyl diphosphate-linked glucose. A VpsA/VpsB/VpsK module analogous to the enterobacterial common antigen machinery synthesizes and transfers an N-acetyl-mannosaminuronic acid (ManNAcA)-derived residue, after which VpsJ, which we propose as a new class of epimerase, catalyzes C5 epimerization to generate the rare bacterial sugar L-N-acetyl-gulosaminuronic acid (L-GulNAcA). Additional tailoring reactions mediated by VpsH, a previously unidentified protein with few sequence or structural homologs, and VpsG introduce glycine and acetyl modifications that are dispensable for repeat-unit assembly but influence matrix properties. Subsequently, glycosyltransfer reactions by VpsI and VpsD complete the tetrasaccharide repeat unit, with VpsD exhibiting substrate flexibility that accounts for the formation of both major and minor VPS variants. Downstream, VpsE and VpsF act following repeat-unit assembly, consistent with flippase and polymerase functions, respectively. Together, our findings establish a molecular framework for VPS assembly and deepen our understanding of the mechanisms that drive biofilm formation in *Vibrio cholerae*.

**IMPORTANCE:**

Biofilm formation is an integral part of *Vibrio cholerae*’s infection cycle, requiring production of the exopolysaccharide Vibrio polysaccharide (VPS). Together, these findings define the sequential enzymatic steps of VPS biosynthesis. This molecular map of VPS production identifies multiple enzymatic nodes as potential anti-biofilm targets and provides a mechanistic foundation for understanding how *V. cholerae* modulates biofilm architecture to enhance environmental survival and transmission.

## INTRODUCTION

*Vibrio cholerae*, the causative agent of cholera, remains a significant global health burden, with an estimated 3–5 million cases and 100,000–120,000 deaths annually worldwide (1, 2). Outside the human host, *V. cholerae* naturally inhabits aquatic ecosystems, where its ability to persist is closely linked to biofilm formation (3–5). Biofilms are structured, surface-associated multicellular communities encased in a self-produced extracellular matrix that enhances bacterial survival under diverse environmental conditions and promotes infectivity (6).

**Fig 1:**
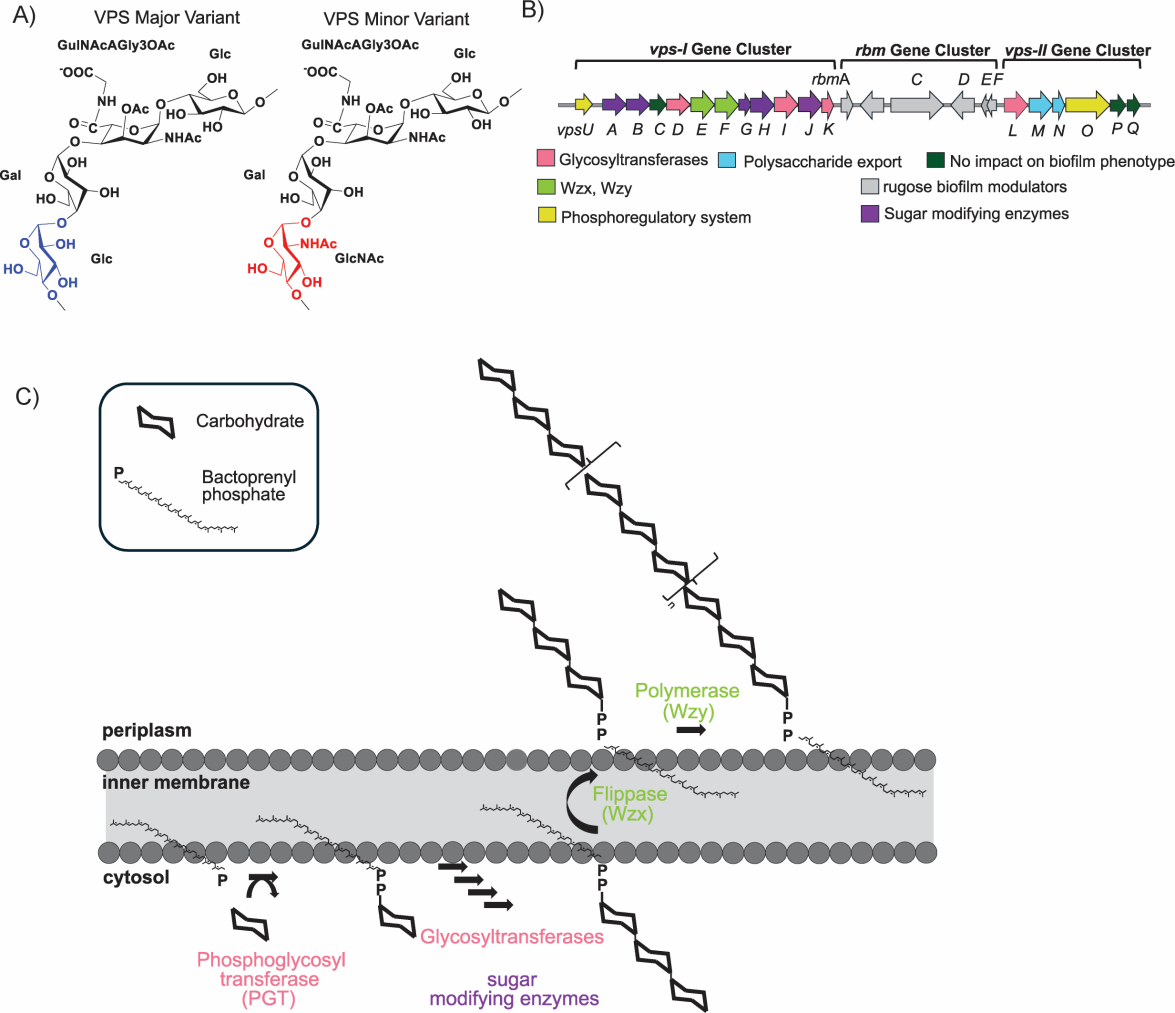
VPS and the Wzx/Wzy polysaccharide biosynthesis system. (**A**) The structure of the major and minor variant repeating units of VPS. (**B**) The vps biosynthesis gene clusters separated by the *rbm* gene cluster. Predicted functions are indicated for each gene. Genes denoted in dark green have no impact on VPS biosynthesis. (**C**) A generic Wzx/Wzy biosynthetic pathway where an initiating phosphoglycosyltransferase (PGT) adds a sugar phosphate to bactoprenyl phosphate, then subsequent glycosyltransferases and sugar-modifying enzymes form the remaining sugars that are then flipped to the periplasm, polymerized, and exported.

A major structural component of the *V. cholerae* biofilm matrix is *Vibrio* polysaccharide (VPS) (Fig. 1), a complex exopolysaccharide that is essential for biofilm formation both in aquatic environments and in hosts (7, 8). Multidimensional NMR structural analyses on isolated VPS have shown that VPS is produced as two related polysaccharide variants that share a tetrasaccharide repeat unit but differ in the identity of one monosaccharide residue (9). The major VPS variant, comprising approximately 80% of isolated VPS, consists of α-D-Gal*p*, β-D-Glc*p*, α-D-Glc*p* (Fig. 1A), and an L-N-acetyl-gulosaminuronic acid with glycine and an acetyl group modification (α-L-GulNAcAGly3OAc). The minor variant consists of an α-D-GlcNAc*p* in place of the α-D-Glc*p*. Although the chemical structures of these polymers are well defined, the molecular mechanisms that govern their biosynthesis, and in particular how *V. cholerae* produces two distinct VPS variants, remain unknown.

In Gram-negative bacteria, surface and secreted polysaccharides are typically synthesized by one of several conserved pathways, including Wzx/Wzy-dependent, ABC transporter-dependent, and synthase-dependent systems (10–12). VPS is thought to be a secreted polysaccharide and has also been observed in association with outer membrane vesicles (13). Based on sequence homology, the VPS biosynthetic machinery is predicted to operate via a Wzx/Wzy-dependent pathway, characterized by the presence of oligosaccharide intermediates assembled on the C55 polyisoprenoid carrier bactoprenyl phosphate (BP or undecaprenyl phosphate [Und-P]), followed by membrane translocation and polymerization (14, 15). In canonical Wzx/Wzy systems, polysaccharide assembly is initiated by a phosphoglycosyltransferase (PGT) that transfers a sugar-phosphate moiety from a nucleotide-sugar donor to BP, after which an ordered series of glycosyltransferases and sugar-modifying enzymes construct the repeat unit prior to translocation across the inner membrane and polymerization (Fig. 1A).

The genes required for VPS biosynthesis and export were originally identified in a VPS-overproducing rugose strain of *V. cholerae*, named for the corrugated colony morphology that reflects enhanced biofilm formation due to enhanced biosynthesis of VPS producing genes (15). These genes are organized into two biosynthetic operons, *vps-I* (*vpsU* and *vpsA–K*) and *vps-II* (*vpsL–Q*) (Fig. 1B), which are separated by the *rbm* (rugose biofilm modulator) gene cluster (15). Several *rbm* genes encode matrix-associated proteins that interact with VPS and are critical for maintaining biofilm architecture (16–18). While genetic and phenotypic studies have established the importance of many *vps* genes for VPS production, the specific biochemical functions of most pathway components and their organization within the biosynthetic pathway remain poorly resolved.

Here, we systematically dissect the VPS biosynthetic pathway in *V. cholerae* by integrating protein sequence analysis and structural prediction with liquid chromatography-mass spectrometry (LC-MS) profiling of isoprenoid-linked VPS intermediates. Building on prior work defining isoprenoid-linked oligosaccharide assembly pathways in *Escherichia coli*, (19, 20) we use targeted *vps* gene deletions to capture and identify pathway intermediates, enabling functional assignment based on the known structure of the polysaccharide and ordering of VPS biosynthetic enzymes. This approach reveals both conserved features shared with other bacterial polysaccharide biosynthesis systems and unique properties important for the generation of two distinct VPS variants, and how it produces the rare bacterial sugar L-GulNAcA with glycine and acetyl group modifications. Together, our findings provide a molecular framework for VPS assembly and advance our understanding of the mechanisms underlying *V. cholerae* biofilm formation.

## RESULTS

### VpsL initiates VPS biosynthesis by forming the first isoprenoid-linked sugar

Wzx/Wzy-dependent polysaccharide biosynthesis pathways are initiated by a PGT that transfers a sugar-phosphate from a nucleotide-sugar donor to the membrane-embedded isoprenoid carrier BP, generating a bactoprenyl diphosphate (BPP)-linked monosaccharide (Fig. 1C) (21). Sequence analysis of the *vps* gene clusters identified four candidate glycosyltransferases, VpsD, VpsI, VpsK, and VpsL, one of which we suspected would function as the initiating PGT. Structural prediction using AlphaFold (22–24) (Fig. S1) revealed that VpsD and VpsI adopt GT-B folds characteristic of GT4 family glycosyltransferases, while VpsK adopts a GT-A fold related to GT26 enzymes based on CAZy database assignments (25). In contrast, VpsL displayed the defining architecture of the monotopic “large” PGT superfamily, including a catalytic core domain, a re-entrant helix, and a membrane-anchoring domain containing four transmembrane helices (21, 26). Sequence and structural comparisons with known and biochemically characterized PGTs (Table 1) (26–28) showed that VpsL is most closely related to the phosphoglucosyltransferases WcaJ and PssY, with >50% sequence identity (Table 1; Fig. S2) and near-identical predicted core structures with a root mean square deviation (RMSD) of 0.353 (WcaJ) and 1.080 Å (PssY) (Table 1; Fig. S3).

**Fig 2:**
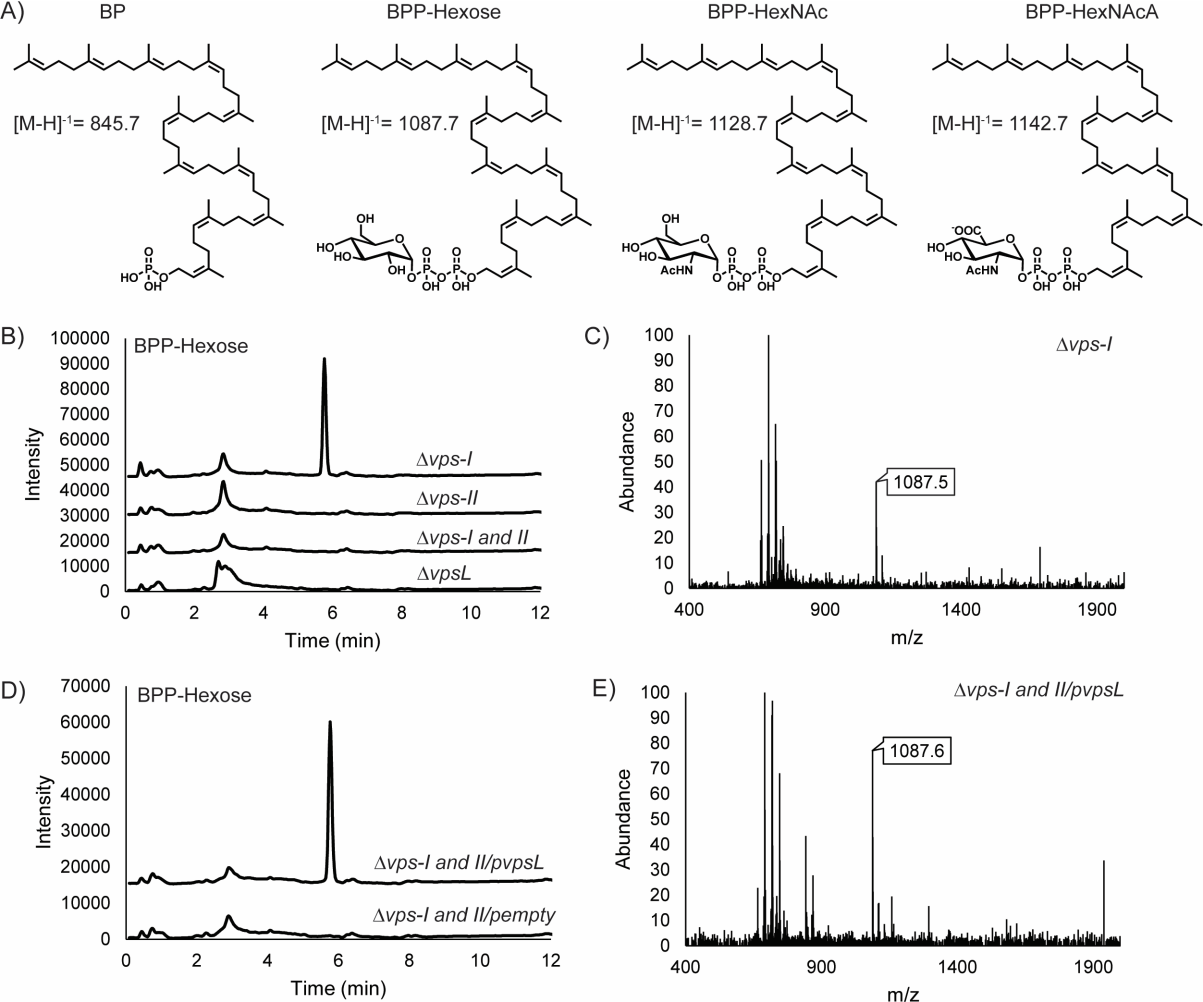
VpsL is the initiating phosphoglycosyltransferase in VPS biosynthesis. (**A**) Structures for possible BPP-linked monosaccharides and BP with [M−H]^−1^ values used for selective ion monitoring. (**B**) Extracted lysates of indicated strains of *V. cholerae* were analyzed by LC-MS in selective ion mode for the *vps-I* and *II* deletion strains combined and separate. Data show the result of BPP-hexose monitoring of the three strains. No peaks were observed for BPP-HexNAc or BPP-HexNAcA (data not shown). (**C**) Mass spectrum of the peak detected in panel B with Δ*vps-I* strain. (**D**) Formation of a BPP-hexose after a plasmid encoding vpsL was transformed into the Δ*vps I-II* strain. (**E**) Mass spectrum of the peak detected in panel D for the vpsL complementation strain.

**Fig 3:**
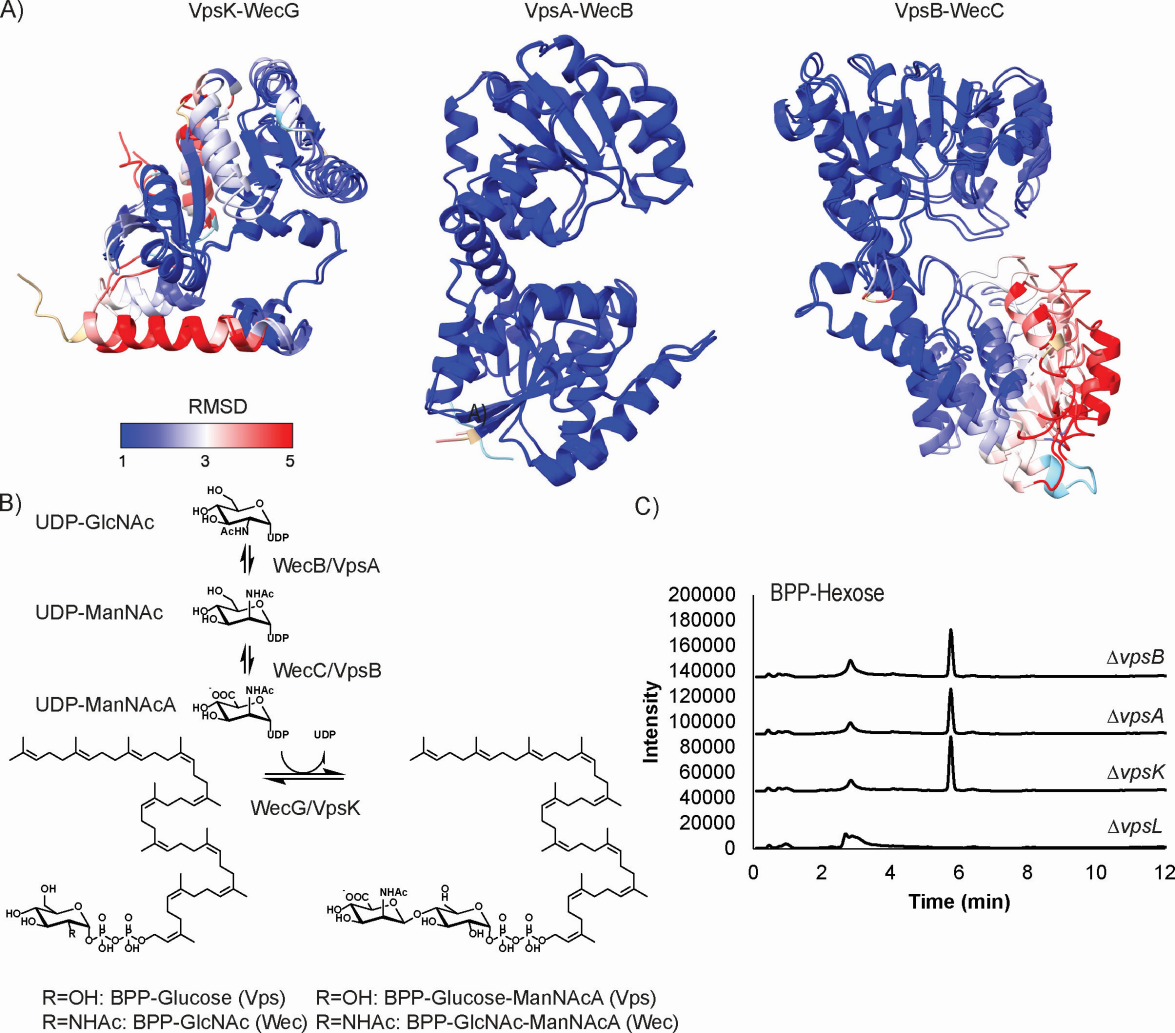
VpsK/A/B form a ManNAcA synthesis and transfer module like the enterobacterial common antigen (ECA) system in *E. coli*. (**A**) Comparison of the predicted structures of VpsK and WecG, VpsA and WecB, and VpsB and WecC color-coded by RMSD. (**B**) Biosynthesis of BPP disaccharide in *E. coli* and proposed analogous system in *V. cholerae*. (**C**) LC-MS analysis of ΔvpsK, ΔvpsA, and ΔvpsB strains compared to ΔvpsL strain in selective ion monitoring (SIM) mode for *m*/*z* 1,087.5 BPP-hexose.

**TABLE 1:** VpsL comparisons with similar function proteins^*a*^

Protein	Organism	UniProt ID	Sugar phosphate specificity	Identity/similarity/gaps (%)	Residues	Predicted structure RMSD (Å)
WcaJ	*Escherichia coli*	P71241	Glc-P	77/88/0	262–464	0.353
Cps2E	*Streptococcus pneumoniae*	Q9ZII5	Glc-P	37/54/9	255–455	4.239
PssY	*Caulobacter vibrioides*	Q9ABR0	Glc-P	50/66/0	65–268	1.080
WbaP	*Salmonella enterica*	S5RN13	Gal-P	41/56/13	270–476	3.468
WecP	*Aeromonas hydrophila*	B3FN88	GalNAc-P	44/62/2	225–423	2.244

^*a*^ Protein blast comparison of VpsL functional core amino acid sequence (UniProtID: Q9KTG8 residues 275–458) with previously characterized bacterial PGTs. Sequence alignments and sequences used are provided in Fig. S2. Predicted structures of the core domains were compared, and RMSD of ɑC pairings is reported for the total alignment. Structure alignments are provided in Fig. S3.

If VpsL is the initiating phosphoglycosyltransferase, we would expect that deletion of this gene would not lead to the accumulation of BP- or BPP-linked intermediates. We tested this using a rugose variant of a clinical *V. cholerae*, O1 El Tor (A1552), as this strain produces ample VPS components and has been used extensively for analysis of matrix components (9, 29–37). Specifically, as with all deletion mutants in this report, we used a rugose variant background of *V. cholerae* carrying deletions of *vpsL* alone, the entire *vps-I* and *II* clusters, or deletion of each gene cluster individually (31, 37). Each strain was grown overnight, pelleted, then lysed and analyzed by LC-MS in selective ion monitoring (SIM) mode targeting BPP-hexose, BPP-N-acetyl-hexosamine (HexNAc), or BPP-N-acetyl-hexosaminuronic acid (HexNAcA), as any of these were possible products of the VpsL-catalyzed reaction (Fig. 2A). LC-MS analysis of BPP-linked intermediates showed that deletion of *vpsL* or the *vps-II* gene cluster (which encodes *vpsL*) led to no accumulation of any BPP-linked intermediates, while deletion of the *vps-I* cluster resulted in accumulation of BPP-hexose (Fig. 2B and C). Expression of *vpsL* alone in a strain lacking both *vps* operons restored BPP-hexose formation, while an empty vector control did not (Fig. 2D and E), demonstrating that VpsL is sufficient to generate the initial BPP-linked hexose, consistent with the similarity of the protein to the phosphoglucosyltransferases WcaJ and PssY. Together, these data establish VpsL as the initiating phosphoglycosyltransferase in VPS biosynthesis.

### VpsK/A/B function analogously to the enterobacterial common antigen WecG/B/C

Based on the VPS repeat unit structure, the second sugar added during biosynthesis could be another hexose, a HexNAc, or HexNAcA. VpsK adopts a GT-A fold and shares sequence (57% sequence similarity, Fig. S4) and predicted structure (RMSD 2.609 Å, Fig. 3A) similarity with the enterobacterial common antigen (ECA) biosynthesis enzyme WecG (20). In addition, the enzymes VpsA and VpsB are closely related by sequence and predicted structure to the ECA biosynthetic enzymes WecB (81% sequence similarity, RMSD 1.684 Å to VpsA) (Fig. 3A; Fig. S4) and WecC (77% sequence similarity, RMSD 2.255 Å to VpsB) (Fig. 3A; Fig. S4). In ECA biosynthesis, WecB and WecC generate UDP-ManNAcA from UDP-GlcNAc, and WecG transfers the ManNAcA sugar to a BPP-linked GlcNAc (Fig. 3B) (20). Deletions of either *vpsK*, *vpsA*, or *vpsB* all led to accumulation of BPP-hexose (Fig. 3C), consistent with VpsK being the first glycosyltransferase acting after VpsL, and loss of the nucleotide-sugar donor required for VpsK activity with deletion of *vpsA* and *vpsB*. These findings indicate that VpsA, VpsB, and VpsK form a functional module analogous to the *E. coli* ECA WecB/WecC/WecG system, generating and transferring an acidic sugar to the growing VPS repeat unit.

**Fig 4:**
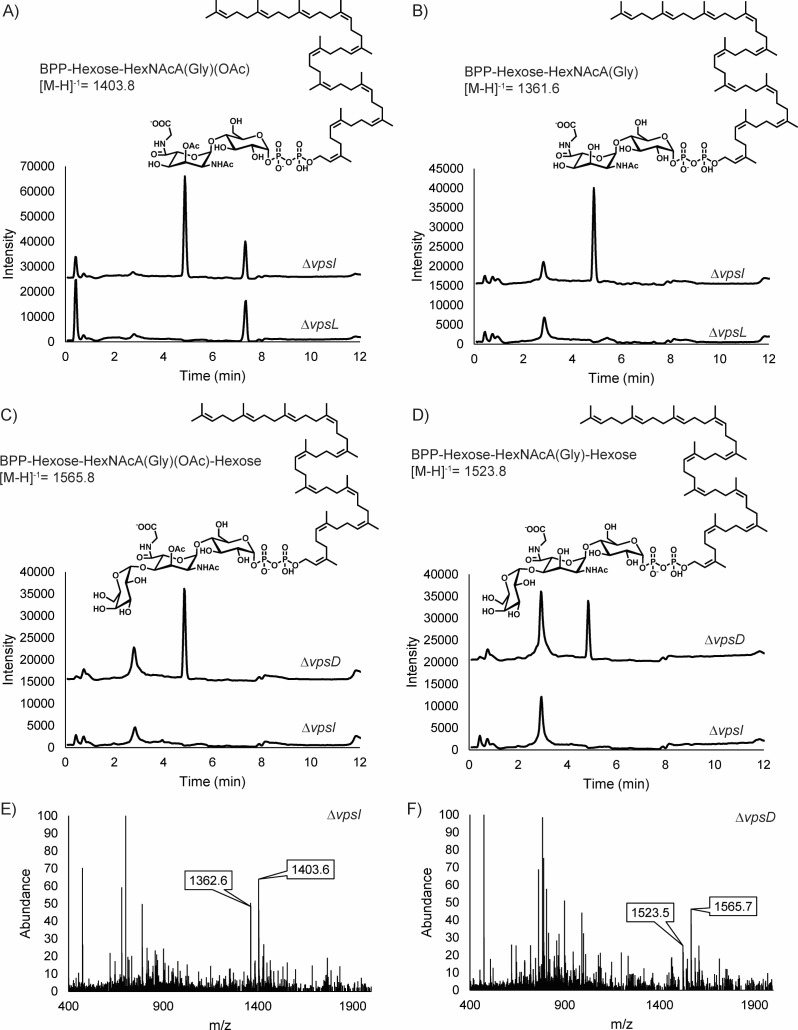
VpsI and VpsD catalyze the second and third glycosyltransferase reactions. SIM chromatogram, structure, and [M−H]^−1^ used for SIM of (**A**) BPP-hexose-HexNAcA(Gly3OAc) and (**B**) BPP-hexose-HexNAcA(Gly) from ΔvpsI and ΔvpsL strains. The unique peak with ΔvpsI is the disaccharide. (**C**) BPP-hexose-HexNAcA(Gly3OAc)-hexose and (**D**) BPP-hexose-HexNAcA(Gly) from ΔvpsD and ΔvpsI strains. The unique peak with ΔvpsD is the trisaccharide. (**E**) Mass spectrum of the unique peak in panel A. (**F**) Mass spectrum of the unique peak in panel C. The presence of the unacetylated disaccharide in the mass spectra is due to overlapping acetylated and unacetylated peaks in the chromatogram.

### VpsI and VpsD catalyze sequential addition of the third and fourth sugars

Following formation of the modified disaccharide, two additional glycosyltransferases, VpsI and VpsD, are required to complete the VPS tetrasaccharide repeat unit. LC-MS analysis of a Δ*vpsI* mutant revealed accumulation of a BPP-linked disaccharide bearing the expected hexose-HexNAcA with glycine and acetyl group modifications, indicating that VpsI catalyzes the addition of the third sugar and that the acetylation and glycine addition occur prior to this step (Fig. 4A). Based on VPS structural data, the sugar added by VpsI is most consistent with galactose. The presence of the HexNAcA in the disaccharide as the second sugar also confirms the first sugar added by VpsL and that VpsK adds the HexNAcA residue. In addition to the formation of the disaccharide with glycine and acetyl group modifications, we also detected a disaccharide without the acetyl group (Fig. 4B).

Deletion of *vpsD* resulted in accumulation of a BPP-linked trisaccharide corresponding to the fully modified three-sugar intermediate (Fig. 4C). Importantly, this intermediate was common to both major and minor VPS variants, indicating that VpsD catalyzes the addition of the final sugar in both cases, as an intermediate would not accumulate if another glycosyltransferase played a role in forming the alternative VPS variant. Like in the Δ*vpsI* strain, we observed a trisaccharide without the acetyl group modification in the Δ*vpsD* strain (Fig. 4D). The expected *m*/*z* for both the VpsI and VpsD substrates with or without the acetyl group was also clear in the mass spectrum for the disaccharide and trisaccharide (Fig. 4E and F). These data suggest that the acetyl group addition may not be required for the activity of VpsI. Altogether, these data establish VpsI and VpsD as the third and fourth glycosyltransferases in VPS biosynthesis and demonstrate that divergence between VPS variants occurs at the level of substrate selection by VpsD rather than through alternative enzymes. These data also suggested that the acetyl group modification is not required for downstream enzyme activity.

### VpsE and VpsF function after assembly of the repeat unit tetrasaccharide

**Fig 5:**
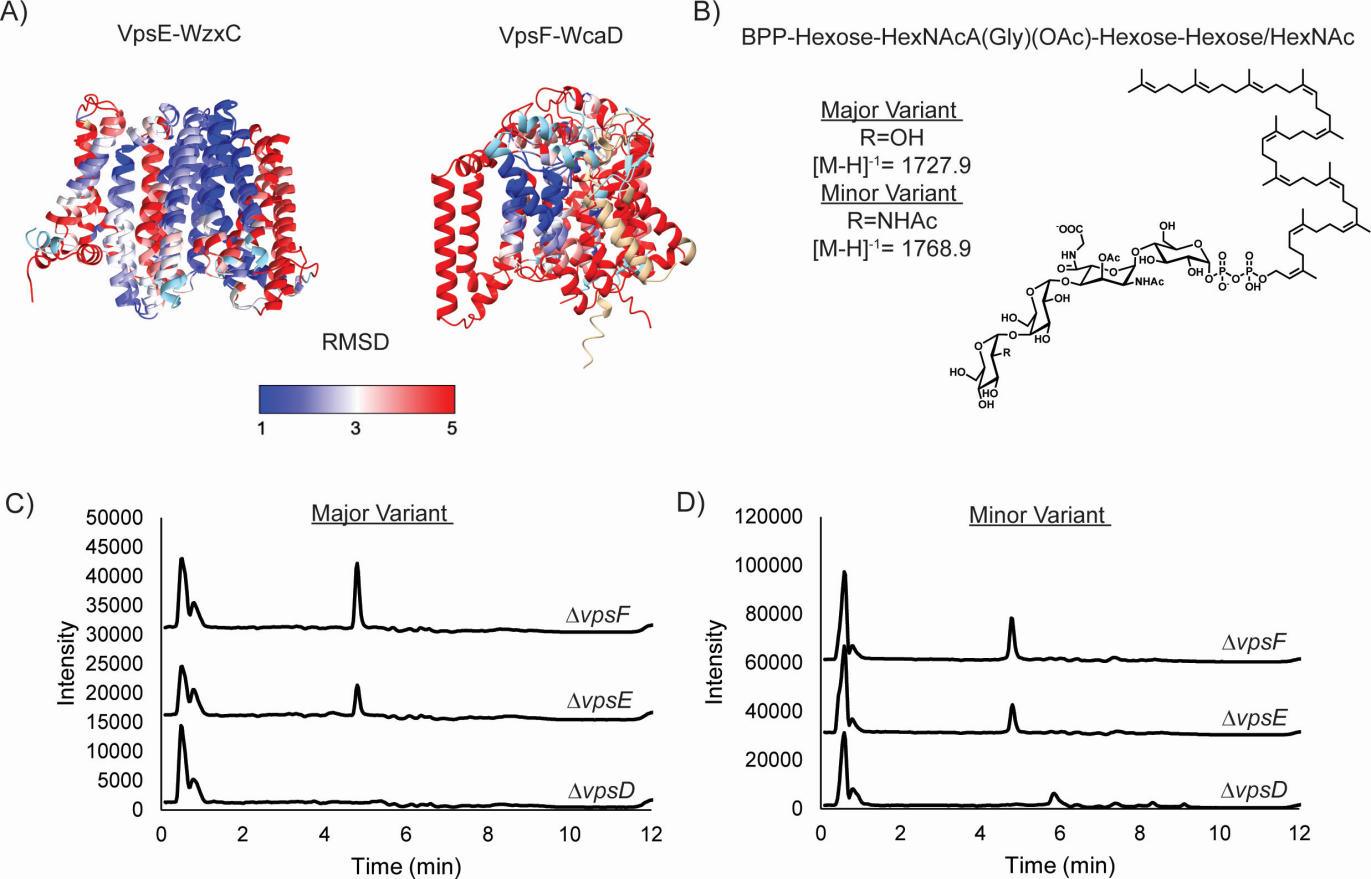
Deletions of VpsE and VpsF result in the accumulation of BPP-tetrasaccharide. (**A**) Structure overlay of VpsE and WzxE of *E. coli* and of VpsF and WcaD of *E. coli* with color-coded RMSD values. (**B**) Structure of VPS major and minor variant repeat unit linked to BPP with appropriate [M−H]^−1^. (**C**) VPS major variant repeating unit detection with both *vpsE* and *vpsF* deletions compared with the *vpsD* deletion strain (**D**) same as panel C except SIM ion detection for the BPP-linked VPS minor variant repeating unit.

VpsE and VpsF are predicted multi-pass membrane proteins similar to Wzx flippases and Wzy polymerases, respectively. Sequence comparisons are typically limited in value for fully embedded membrane proteins; therefore, we compared the predicted structures of VpsE and VpsF with a known flippase and polymerase (Fig. 5A). We found that VpsE is structurally similar to WzxE (flippase) (RMSD 4.71 Å) of the ECA pathway in *E. coli*, whereas the best fit we could find for VpsF was WcaD (polymerase) (RMSD 11 Å) of the colanic acid pathway in *E. coli*; however, that structural similarity was limited. Consistent with these assignments, deletion of either *vpsE* or *vpsF* led to accumulation of fully assembled BPP-linked tetrasaccharide repeat units corresponding to both major and minor VPS variants (Fig. 5B through D). These results suggest that VpsE and VpsF function after the assembly of the tetrasaccharide repeat unit, consistent with their assignments as the flippase and polymerase, respectively.

### VpsJ is required for L-GulNAcA formation, consistent with a C5 epimerase function

**Fig 6:**
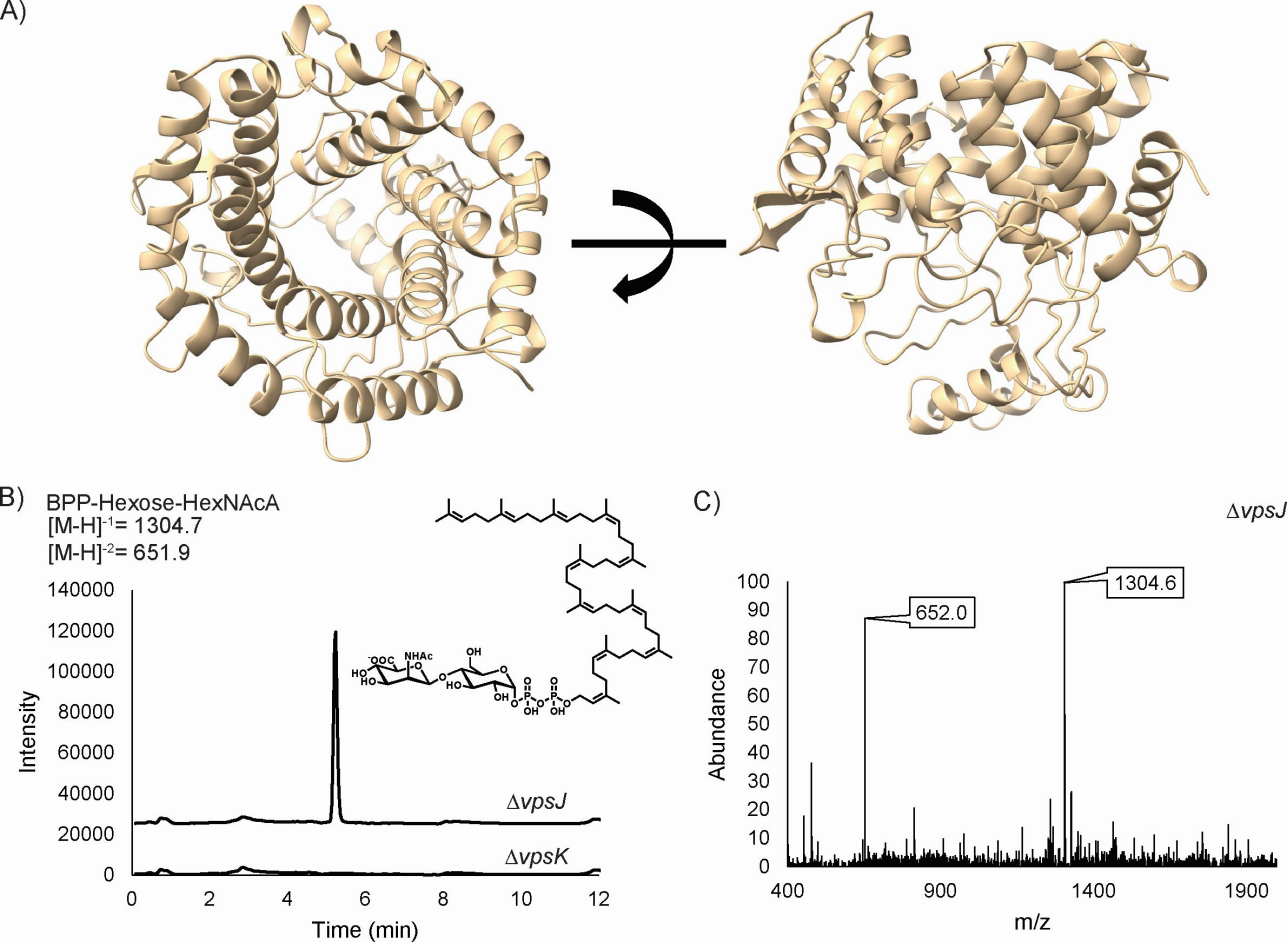
VpsJ is a potential C5 epimerase. (**A**) AlphaFold predicted structure of VpsJ. (**B**) Structure, [M−H]^−1^ and [M−2H]^−2^ and SIM chromatograms of the proposed substrate for VpsJ BPP-Glc-ManNAcA in a Δ*vpsJ* and Δ*vpsK* strain. (**C**) Mass spectrum of the unique Δ*vpsJ* peak detected in panel B.

With the deletion of *vpsI,* we only detected an intermediate that included a glycine modification. It was not clear whether this modification is required for VpsI to function and which protein is responsible for the modification. Sequence searches on VpsJ did not indicate a similar sequence to a protein with an obvious function in VPS assembly. However, analysis of the AlphaFold predicted structure of VpsJ (Fig. 6A) using FoldSeek (38) resulted in the detection of similar folds in a series of epimerases, including a GlcNAc-2-epimerase (Fig. S5). Deletion of *vpsJ* resulted in accumulation of only an unmodified BPP-linked disaccharide, indicating that VpsJ acts after VpsK but before glycine and acetyl group modifications (Fig. 6B and C). Based on these data, we propose that VpsJ catalyzes C5 epimerization of D-ManNAcA to L-GulNAcA. This reaction generates the substrate required for downstream modifications (glycine and acetyl group additions) and glycosyltransfer reactions.

### VpsH catalyzes transfer of a glycine unit to VPS

**Fig 7:**
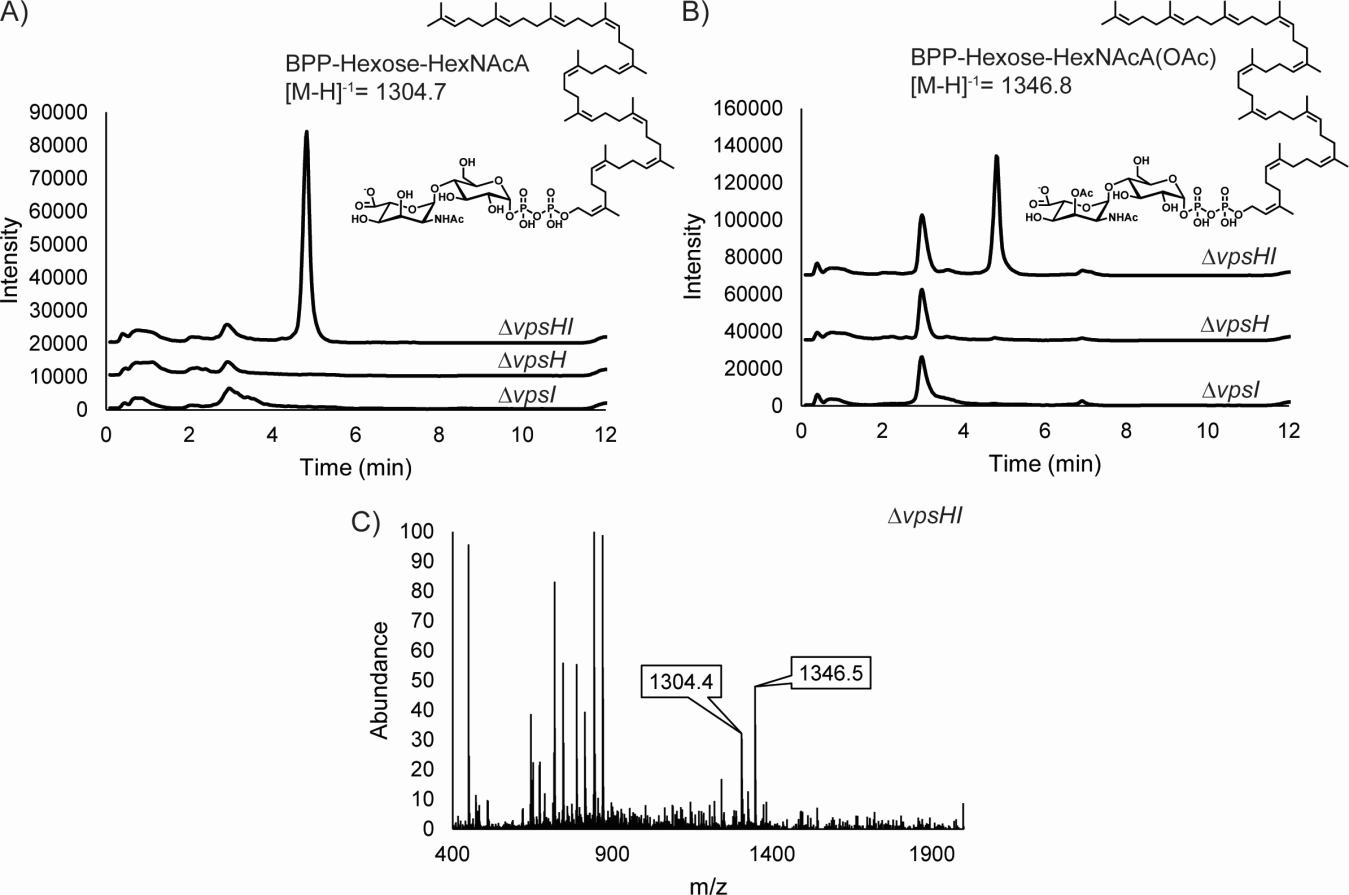
VpsH catalyzes the addition of glycine to the VPS disaccharide. (**A**) Structure and [M−H]^−1^ and SIM ion detection of BPP-hexose-HexNAcA in the double mutant Δ*vpsH*Δ*vpsI*, Δ*vpsH*, and Δ*vpsI*. (**B**) Structure of proposed BPP-glucose-GulNAcA(OAc) substrate of VpsH. (**C**) Mass spectrum of the unique Δ*vpsH*Δ*vpsI* peak shown in panel A that contains both the unacetylated and acetylated forms of disaccharide VPS intermediate.

The *vpsH* gene was previously shown to decrease colony corrugation but still resulted in biofilm pellicle formation (15). VpsH was predicted to be related to a phenylacetate CoA ligase. However, there was no indication that such a protein would be useful for polysaccharide production. In addition, sequence and structure similarity between VpsH and a phenylacetate CoA ligase from *E. coli* was limited (Fig. S6) with only 27% sequence identity and 40% similarity and an RMSD of the predicted structure overlays of 9.276 Å. However, 154 residues were within 1.281 Å suggesting some potential mechanistic overlap with VpsH. With deletion of *vpsH* we did not observe the accumulation of any intermediates. We considered that VpsH may be responsible for glycine addition and that this modification may not be required for further processing by VpsI and other downstream enzymes. If that is the case, then deletion of *vpsH* in a Δ*vpsI* background could lead to the trapping of the VpsH substrate. We prepared the double mutant of *vpsH* in a Δ*vpsI* background and analyzed extracted lysate for accumulation of an intermediate disaccharide without the addition of a glycine unit. We found a glycine-free disaccharide with and without the acetyl group modification (Fig. 7A and B). Inspection of the mass spectrum from the LC-MS peaks further confirmed the presence of these materials (Fig. 7C). These data suggest that acetylation may occur before or after glycine addition.

### VpsG transfers an acetyl group to VPS

**Fig 8:**
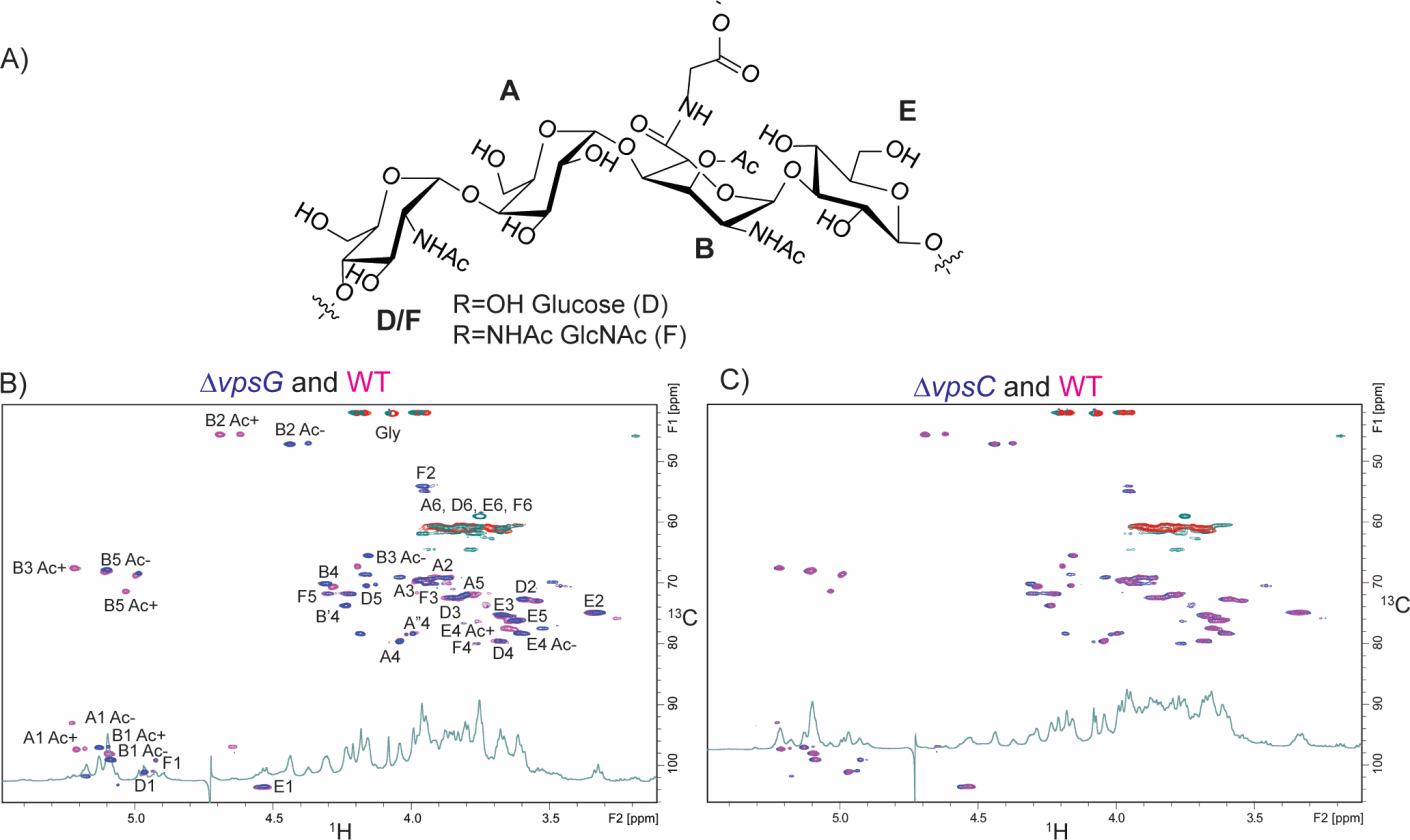
VpsG and not VpsC impact acetylation of VPS. (**A**) Vps structure and letter designation for assignments. VPS was isolated from Δ*vpsC*, Δ*vpsG*, and WT strains and then analyzed by ^1^H^13^C HSQC of VPS isolated from (**B**) Δ*vpsG* (blue [CH, CH_3_ phase up]/green [CH_2_ phase down]) and wild type (pink [CH, CH_3_ phase up]/red [CH_3_ phase down]) or (**C**) Δ*vpsC* (blue [CH, CH_3_ phase up]/green [CH_2_ phase down]) and wild type (pink [CH, CH_3_ phase up]/red [CH_3_ phase down]). Note the presence of unacetylated VPS in both strains, but the absence of O-acetylation in the Δ*vpsG* strain. A ″4 is from the minor variant galactose, where the 4-position proton is impacted by the NHAc group of GlcNAc relative to the OH group of glucose.

Two genes were predicted to encode an acetyltransferase, *vpsC* and *vpsG*, and are located within the first VPS biosynthesis gene cluster. Both VpsC and VpsG sequences were greater than 50% similar to the *E. coli* acetyltransferase WcaB (Fig. S7). The predicted structures of VpsC and VpsG were only weakly related to the predicted structure of WcaB at an RMSD of 8.485 and 7.550 Å, respectively (Fig. S7). However, the characteristic β-sheet region of these proteins was very closely related to WcaB, where over 60% of residues were within an RMSD of 0.646 and 0.739 Å for VpsC and VpsG to WcaB. Previous work demonstrated that deletion of *vpsG* led to an intermediate colony corrugation phenotype, while deletion of *vpsC* had no impact on biofilm-related phenotypes of *V. cholerae*. (15) We expected a Δ*vpsC* strain not to accumulate BPP-linked intermediates and for a Δ*vpsG* strain to accumulate a BPP-hexose-HexNAcA modified with just a glycine. LC-MS analysis of these strains indicated that no BPP-linked intermediates accumulated in either strain, and attempts to trap an intermediate with deletion of *vpsG* in a *vpsI* background were unsuccessful (data not shown). VPS was isolated from Δ*vpsG* and Δ*vpsC* strains and compared with VPS isolated from the rugose strain. ^1^H–^13^C HSQC analysis of the isolated VPS was then compared among the three strains (Fig. 8). The VPS isolated from the Δ*vpsC* strain had no changes in acetylation patterns relative to VPS isolated from the rugose strain, while VPS isolated from the Δ*vpsG* strain indicated a loss of acetyl groups relative to the other two strains. Close inspection of the HSQC data demonstrated peak shifts for the L-GulNAcA proton/carbon correlations at the 1, 2, 3, and 5 positions and peak shifts for the glucose (E, Fig. 8A) at the 4-position, and Gal (A, Fig. 8A) 1-position, both of which are in close proximity to the expected acetylation site at the 3-position of the L-GulNAcA residue. These results demonstrate that VpsG catalyzes acetylation of VPS and that this modification is dispensable for repeat unit assembly. It is still not clear why the acetyl group-free intermediate was not trapped in the Δ*vpsG* strain in the Δ*vpsI* background.

## DISCUSSION

**Fig 9:**
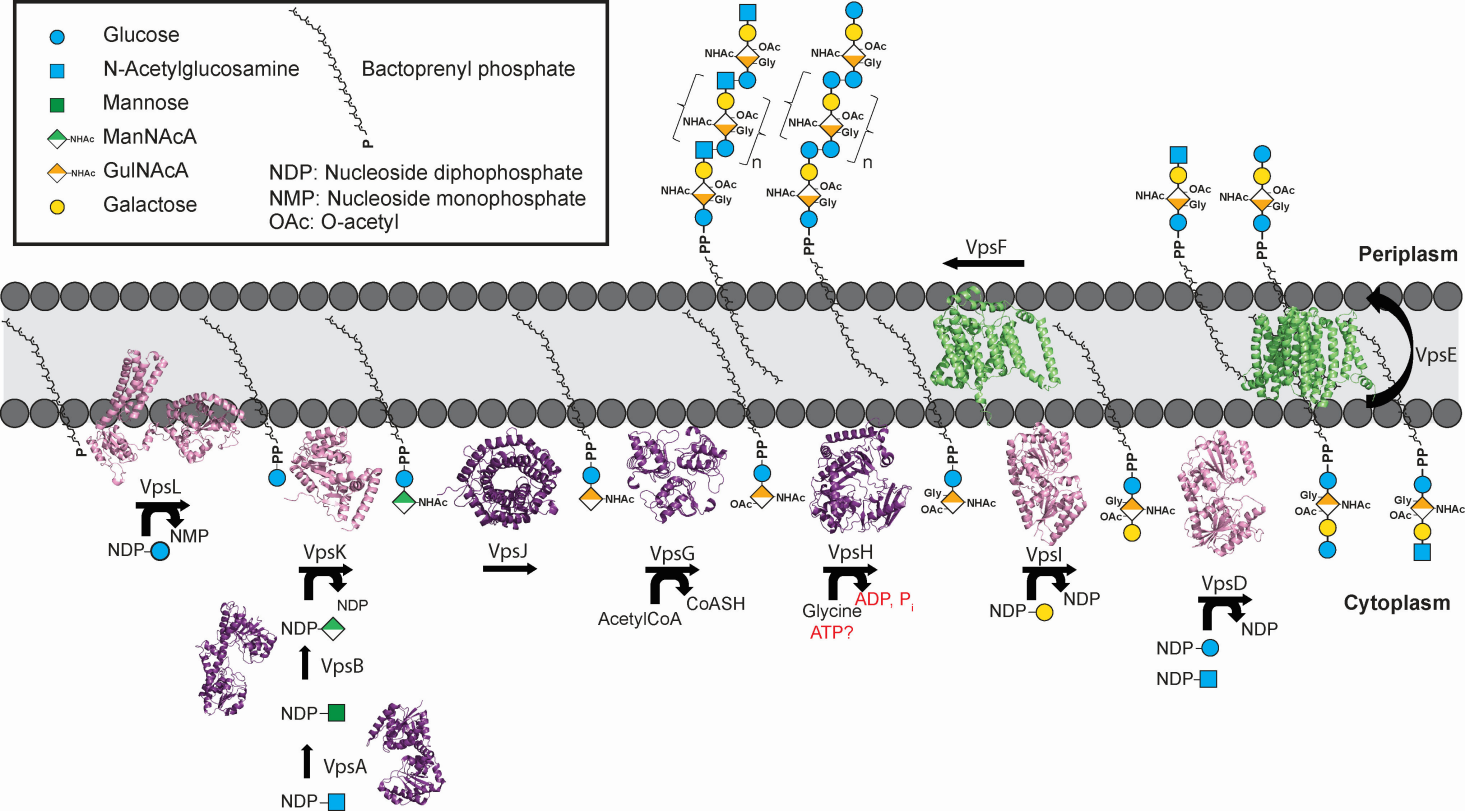
Model pathway for VPS biosynthesis by *Vibrio cholerae*.

Biofilm formation is central to the environmental persistence and transmission of *Vibrio cholerae*. The VPS constitutes the dominant structural component of the mature biofilm matrix, yet the biochemical logic underlying its assembly has remained incomplete. Here, by integrating genetic analysis, structural modeling, and profiling of isoprenoid-linked intermediates, we define an ordered Wzx/Wzy-dependent pathway for VPS biosynthesis (Fig. 9). Our findings assign biochemical functions to the majority of Vps proteins, establish the sequence of repeat unit assembly on the BP carrier, and reveal how *V. cholerae* generates two structurally distinct VPS repeat variants within a single biosynthetic framework.

### VPS initiation and conservation of phosphoglycosyltransferase function

Our data support a model in which VpsL functions as the initiating phosphoglycosyltransferase, catalyzing transfer of glucose-1-phosphate to BP, generating BPP-Glc. VpsL shares clear structural similarity with WcaJ from *Escherichia coli*, *Salmonella enterica*, and *Klebsiella pneumoniae*, the initiating enzyme of colanic acid biosynthesis. Structural comparisons using Foldseek identified additional highly significant similarity to characterized BP glucose-1-phosphate transferases, including GumD from *Xanthomonas campestris*, as well as PslA from *Pseudomonas aeruginosa*. This degree of conservation is consistent with the central role of the initial lipid-linked monosaccharide in Wzx/Wzy-dependent assembly pathways.

### The VpsA/B/K module and parallels to ECA biosynthesis

We define a UDP-ManNAcA biosynthesis and transfer module consisting of VpsA, VpsB, and VpsK that parallels the ECA pathway in Enterobacterales. Structural comparisons of VpsA reveal strong similarity to characterized UDP-GlcNAc 2-epimerases (WecB-like enzymes) across diverse taxa, including Gram-positive and Gram-negative organisms. However, as previously illustrated by the inability of *E. coli* WecB to complement *P. aeruginosa* WbpI, structural similarity alone does not ensure functional interchangeability (39). In contrast, structural homologues of VpsK were far less prevalent, suggesting a narrower phylogenetic distribution. Together, the VpsA/B/K module appears most conserved among Enterobacterales and select Gram-positive organisms, such as *Staphylococcus aureus*, suggesting either horizontal acquisition or selective retention of this module in organisms producing ManNAcA-derived surface polysaccharides.

### C5 epimerization and formation of L-GulNAcA

One of the most unusual features of VPS is the presence of L-GulNAcA, a rare sugar found in only a limited number of bacterial glycans. We propose that VpsJ catalyzes C5 epimerization of ManNAcA after its incorporation into the BPP-linked intermediate. While distant similarity exists to 2-epimerases and aspartate-semialdehyde dehydrogenases, sequence identity is low, and RMSD values are substantial for predicted structure overlays. Nevertheless, catalytic residues implicated in acid-base chemistry in a known cellobiose-2-epimerase (3WKF) that cluster in analogous regions of VpsJ raise the possibility of convergent active-site architecture (Fig. S8).

VpsJ appears to be most closely associated with aspartate-semialdehyde dehydrogenases. C5 epimerization in sugar nucleotide metabolism is rare. Alternative dehydrogenase-mediated epimerization mechanisms, such as those proposed for WbjB-dependent pathways, suggest that transient oxidation could facilitate proton abstraction at C5 (40). We speculate that oxidation at C4 and/or activation by the carboxylic acid at C6 could increase the acidity of the C5 proton, enabling stereochemical inversion. Direct biochemical characterization of VpsJ will be required to resolve this mechanism.

### Amino acid modification and VpsH

VpsH appears to mediate amino acid modification of the VPS repeat unit. Amino acid substitutions on bacterial polysaccharides are uncommon but have been reported in capsules of *S. aureus* (41), *Bacillus anthracis* (42), and select *E. coli* K antigens (43–45). Structural comparisons suggest partial similarity of VpsH to CapK-like proteins and to acyl-CoA ligase families, raising the possibility of ATP-dependent activation of a carboxylate prior to amide bond formation. However, VpsH lacks strong sequence or structural similarity to previously characterized amino acid ligases involved in capsule modification, including the K40/K54 serine transferases or Dapdiamide ligases that appear to be the most structurally related (46). Thus, VpsH may represent a distinct mechanistic solution for amino acid incorporation into polysaccharides. Biochemical characterization will be required to determine whether VpsH operates through acyl-phosphate, adenylate, or alternative activation chemistry. Importantly, deletion of *vpsH* or *vpsG* does not lead to accumulation of lipid-linked intermediates, indicating that glycan extension by VpsI can proceed independently of these modifications. This suggests that amino acid decoration is not required for polymer elongation but instead modulates biofilm architecture or matrix properties, and this is consistent with previous observations of colony morphology of *vpsG* and *vpsH* deletion mutants (15). In these mutants, *vpsH* deletion leads to a smooth colony phenotype like strains that do not produce VPS. However, *vpsH* mutants can form pellicle biofilms. Alternatively, deletion of *vpsG* leads to an intermediate colony corrugation phenotype. This previous work indicated that these genes are important in biofilm formation, and this work indicates that these impacts are likely due to loss of glycine or acetyl group modifications. Future work will focus on precisely how these modifications impact functional properties of VPS or whether phenotypic changes previously observed are due simply to a change in VPS quantities when these modifications are not present.

### VpsI and VpsD: GT-B enzymes and repeat unit diversification

VpsI and VpsD both are predicted to belong to the GT4 family and adopt GT-B folds composed of two Rossmann-like domains. Predicted structural comparisons reveal partial similarity to GlcNAc-malate transferases, such as BshA, from *B. anthracis* and *S. aureus* (47–49). Although these enzymes catalyze transfer to acidic acceptors rather than canonical sugars, this may be relevant given the acidic L-GulNAcA residue within the VPS acceptor substrate.

VpsD is responsible for the formation of both major and minor VPS repeat variants. Such donor or acceptor promiscuity is uncommon among bacterial glycosyltransferases involved in glycopolymer biosynthesis, which are typically highly specific. Promiscuous GT-B enzymes have been described in antibiotic modification pathways (e.g., OleD, GtfA/B) (50, 51), oligosaccharyl transferases (PglL) (52), and certain sialyltransferases (53). VpsD represents, to our knowledge, the first example of a glycosyltransferase performing two biologically relevant repeat-unit modifications within a capsular assembly pathway. The ability of *V. cholerae* to generate VPS heterogeneity through controlled glycosyltransferase promiscuity may provide adaptive flexibility in biofilm structure, immune evasion, or environmental persistence. This modular yet flexible architecture underscores how incremental enzymatic diversification can generate structural complexity without requiring wholesale reorganization of the biosynthetic pathway. How the polymerase distinguishes and controls polymerization of the same repeat units without the formation of a mixed polymer is still unclear and requires further investigation of the polymerase and other proteins that may be involved in polymerization.

### Conclusions

The ordered logic of VPS assembly revealed here identifies multiple enzymatic nodes that are attractive targets for anti-biofilm intervention. Initiating phosphoglycosyltransferases, such as VpsL, represent bottlenecks in lipid-linked assembly, and inhibition would prevent the formation of all downstream intermediates. Similarly, the VpsA/B/K module and the proposed C5 epimerase, VpsJ, introduce chemically unique transformations not found in human metabolism, increasing the likelihood of selective inhibition. Because VPS is essential for mature biofilm architecture and environmental persistence of *V. cholerae*, targeting its assembly could disrupt colonization and transmission without directly inhibiting bacterial growth, potentially reducing selective pressure for resistance (3). The modularity of the Wzx/Wzy pathway further suggests that inhibitors developed against conserved initiation or transfer reactions may have broader activity against related biofilm-forming pathogens.

## MATERIALS AND METHODS

### Bacterial strains and culture conditions

All bacterial strains and plasmids used in this study are listed in Table S1. *E. coli* DH5α λ *pir* was used for DNA and plasmid construction and manipulation. *E. coli* S17 λ *pir* was used for conjugation with *V. cholerae. E. coli* and *V. cholerae* strains were grown aerobically in Luria-Bertani (LB) broth (1% tryptone, 0.5% yeast extract, 1% NaCl, pH 7.5) at 37°C and 30°C, respectively, with 200 rpm shaking. Granulated agar (Difco) was added at 1.5% (wt/vol) for LB agar media. Medium additives, when necessary, were used at the following concentrations: 100 μg/μL rifampicin (Rif); 100 μg/μL ampicillin (Amp); 100 μM IPTG. All overnight *V. cholerae* liquid cultures were obtained by inoculating five single colonies from LB agar plates into 5 mL LB media.

### Strain and plasmid construction

Plasmids were constructed using standard cloning methods or the Gibson Assembly recombinant DNA technique (New England BioLabs, Ipswich, MA). Restriction enzymes were purchased from New England Biolabs.

Gene deletions were carried out using allelic exchange of the native open reading frame (ORF) for the truncated or extended ORF, as previously described (54). Polymerase chain reactions were carried out with primers purchased from Integrated DNA Technologies and Q5 High-Fidelity master mix (New England BioLabs). The sequencing, performed by Genewiz/Azenta Life Sciences (Chelmsford, MA), was used for construct verification. All *V. cholerae* sequences were amplified from genomic DNA isolated from the *V. cholerae* A1552 strain.

For the overexpression construct, an open reading frame of the *vpsL* gene was cloned into pMMB67.EH vector, which contains the IPTG inducible Ptac system. Resulting plasmid was transformed into the *E. coli* S17-1 λ pir donor strain and introduced into the *V. cholerae* A1552 strain by conjugation.

### Preparation of bacterial cell lysates

*V. cholerae* strains were grown on LB Agar medium at 30°C overnight (18 h). A single colony was picked and used to inoculate a 5 mL LB culture, shaken at 30°C overnight at 220 RPM. Cells were pelleted at 7,870 × *g* for 5 min, then washed with 0.9% NaCl. The supernatant was removed, and cells were resuspended in 700 μL of autoclaved water and transferred to a glass tube. Next, 3 mL of methanol/chloroform (2:1) was added. The solution was vortexed and centrifuged at low speed to remove insoluble debris. Soluble supernatant was transferred to new glass tubes and placed at −80°C until a slurry was formed, and tubes were placed on a centrivap to dry under vacuum. The vacuum-dried crude cell lysate was resuspended in 200 μL of 1:3 *n*-propanol and 0.1% ammonium hydroxide and stored at −20°C for up to 1 week.

### LC-MS analysis

Cells from a minimum of three replicates for each were resuspended in 1:3 *n*-propanol, 0.1% ammonium hydroxide and were sonicated for 3 min, and centrifuged at 14,100 × *g* for 5 min. Twenty microliters of clarified lysate was injected and analyzed on an Agilent 1260 Infinity II system equipped with a single quadrupole electrospray ionization MS detector. Analysis was performed using a C18 BEH Waters column at 1 mL/min under gradient conditions starting with 5% *n*-propanol/95% 0.1% ammonium hydroxide, and *n*-propanol was increased over 8 min to 95%. All MS analysis was performed in negative ion mode with one channel devoted to an *m*/*z* range of 400–2,000 with a scan rate of 200 units/s, and the other three channels were utilized for specified selected ions. The source voltage was 4,000 V, capillary temperature 350°C, with a flow rate of 12 L/min and nebulizer pressure of 50 psig.

### Protein sequence acquisition

FASTA-format amino acid sequences of VPS proteins were retrieved from the NCBI Protein Database (https://www.ncbi.nlm.nih.gov/protein). Each sequence was manually curated to ensure accuracy and completeness prior to analysis and used for protein sequence alignment and protein localization predictions.

### Structural alignment of VPS biosynthesis proteins using ChimeraX

VPS protein structures and other proteins used in comparison were downloaded in PDB format from Uniprot protein structure database, which included AlphaFold predicted structures, and were aligned in ChimeraX 1.10.1 (55–57). The structures were inspected, ensuring correct orientation and completeness. Superimposition was carried out using the command Matchmaker, and RMSD values were calculated as part of the matchmaker output to assess the degree of structural similarity and how well they are aligned.

### Protein sequence alignment using BLASTp

Pairwise protein sequence comparison was performed using BLASTp (v2.13.0) against the NCBI non-redundant protein database. The two query sequences were aligned using default parameters, including the BLOSUM62 substitution matrix and standard gap penalties. An E-value cutoff of 1 × 10⁻⁵ was applied to assess the significance of alignments. Sequence identity, similarity, and alignment coverage were calculated to evaluate homology and functional relatedness between the protein sequences.

### ^1^H–^13^C HSQC characterization of acetylation state of VPS

VPS preparation and 2D NMR were performed as described previously (9, 58).

## SUPPLEMENTAL MATERIAL

Supplemental material mbio.01096-26-s0001.pdf Supplemental figures and tables.

ASM does not own the copyrights to Supplemental Material that may be linked to, or accessed through, an article. The authors have granted ASM a non-exclusive, world-wide license to publish the Supplemental Material files. Please contact the corresponding author directly for reuse.
