# Supplementary material for: Ordered assembly of the Vibrio cholerae biofilm exopolysaccharide defined by lipid-linked intermediate profiling
Source: mBio. 2026 Jul 14;17(8):e01096-26. doi: 10.1128/mbio.01096-26 (PMC13463779; doi:10.1128/mbio.01096-26)
Supplement: Supplementary file 1 [file mbio.01096-26-s0001.pdf]

**Supporting Table 1:** List of strains and plasmids used in this study

| Strain or plasmid | Relevant genotype | Source |
|---|---|---|
| ***E. coli* strains** | | |
| DH5-α λ pir | F' *endA1 hsdR17 supE44 thi-1 recA1 gyrA96 relA1* Δ(*argF-lacZYA*) U169(Φ80*lac*D*M15*) λ *pir* | BCCM Gene corner |
| S17-1 λ *pir* | $Tp^r$ $Sm^r$ *recA thi pro* $rK^-$ $mK^+$ RP4::2-Tc::MuKm Tn7 λ *pir* | (1) |
| ***V. cholerae* strains** | | |
| FY_Vc_2 | *Vibrio cholerae* O1 El Tor A1552, Rugose variant, $Rif^r$ | (2) |
| FY_Vc_234 | FY_Vc_2 Δ*vpsI* (VC0916-27) | (3) |
| FY_Vc_4324 | FY_Vc_2 Δ*vpsII* (VC0934-39) | (4) |
| FY_Vc_4327 | FY_Vc_2 Δ*vpsI/II* (VC0916-27; VC0934-39) | (4) |
| FY_Vc_3380 | FY_Vc_2 Δ*vpsL* | (4) |
| FY_Vc_19687 | FY_Vc_4327 pMMB67.EH-*vpsL* | This work |
| FY_Vc_3377 | FY_Vc_2 Δ*vpsK* | (4) |
| FY_Vc_2784 | FY_Vc_2 Δ*vpsA* | (4) |
| FY_Vc_3682 | FY_Vc_2 Δ*vpsB* | (4) |
| FY_Vc_3374 | FY_Vc_2 Δ*vpsI* | (4) |
| FY_Vc_3685 | FY_Vc_2 Δ*vpsJ* | (4) |
| FY_Vc_4976 | FY_Vc_2 Δ*vpsH* | (4) |
| FY_Vc_3326 | FY_Vc_2 Δ*vpsG* | (4) |
| FY_Vc_19689 | FY_Vc_2 Δ*vpsH* Δ*vpsI* | This work |
| FY_Vc_19895 | FY_Vc_3374 Δ*vpsG* | This work |
| FY_Vc_2787 | FY_Vc_2 Δ*vpsD* | (4) |
| FY_Vc_4974 | FY_Vc_2 Δ*vpsE* | (4) |
| FY_Vc_4951 | FY_Vc_2 Δ*vpsF* | (4) |
| **Plasmids** | | |
| pGP704-*sacB*28 | pGP704 derivative, *mob*/*oriT sacB*, $Amp^r$ | G. Schoolnik |
| pFY_7862 | pGP704-sacB28::Δ*vpsHI*, $Amp^r$ | This work |
| pMMB67.EH | Expression vector harboring *lacI* and containing a Ptac promoter, $Amp^r$ | (5) |
| pFY_7863 | pMMB67.EH-*vpsL*, $Amp^r$ | This work |

**Supporting Figure 1.** Sequences and alphafold predictions of each annotated glycosyltransferase in VPS gene clusters *I* and *II*.

**VpsD**

MSKILLIMPLSTLNWGEKNLGGVDSVCQMLVRQLAQQERPYHYRVLAFDPLNNHSYSGEIIQLSK
HLEVVICPLREKRFGLPLPSLLSNWLRIQEQLKDYQPDLVHSHLNSWMMGLGQKTRNVLTLHSY
RKIGRKPVSKLNDFVYEQIIPWVSHFSVDFYTCVGEELRQALSLETNKSIQVIGNPVDPDYFSANS
ANQNLPQNEVNLVTCALITRRKRIDRAIVLLRELKQRGQAATLRIIGPNMDSAYYAQLQQLIKEYE
LEQDVIFLGKLNQREIVQQYQQANIGIFTSQQETFGLAPLEMMAAGLPLISTPVGILGERQATFDQ
LGVVFMQEGQEAMIAERISQIKITDTQAIRTYLRDQFAVENVIEHYQNLYREVLS

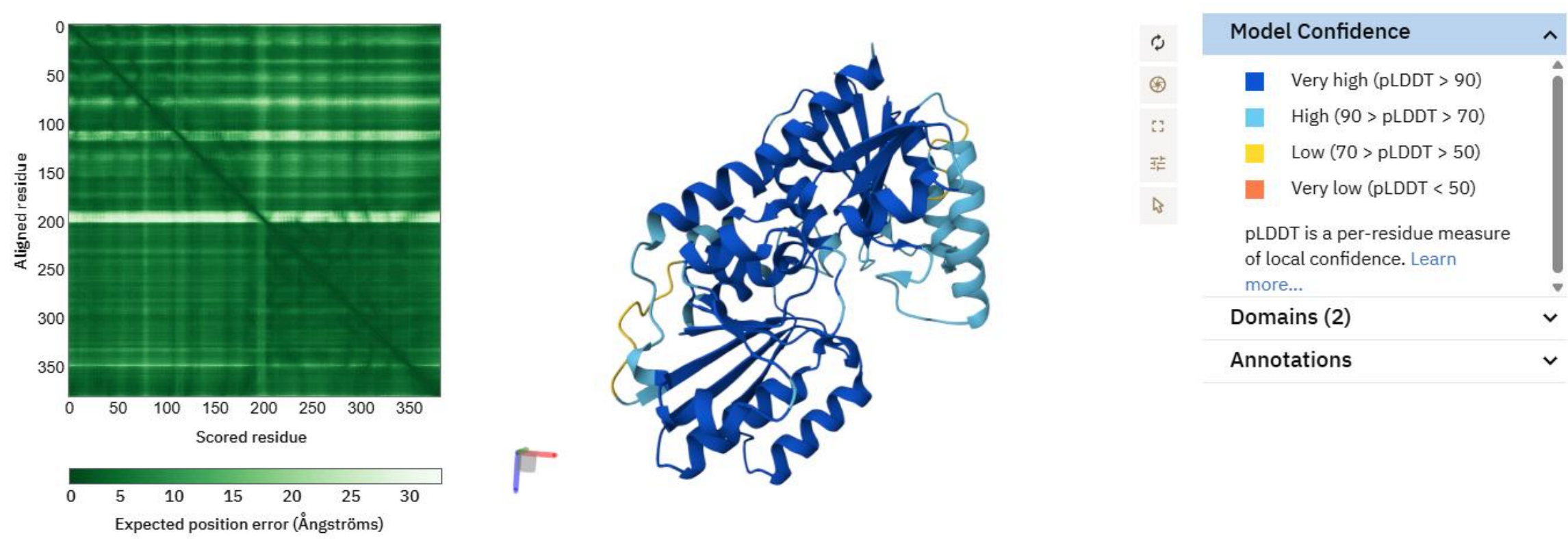


**VpsI**

MRKNINLMIATDLKGQGGVATVVSTYADCGFLEENHIKLIASHSSLDHGQKWRMTLRFMLALCQ
LLYCFMRYRVGIVHIHMSSRGSYRRKSVIIRLVKLLKGKVILHLHGAEFRDFYRDECNMVQQRHI
RHTFALADHVLVLSTQWLAWLQEVIGRTQGVSVLYNAVPSLALDRHLAQPGRIAFLGRLGTRKG
VGDLIQAFALVKQRCPEAQLYLAGDGEIETYQAMAEQLGLNGSVHCLGWIAGEAKLKLLTQTDI
YCLPSYNEGFPMGVIEAMSAGIPVVASRAGGIPDAISDGEQGRLIEAGDVVALAQALGDLIEQRAE
NQRIATAAKQKFAENFSLQAVIPRLQTLYDELLKS

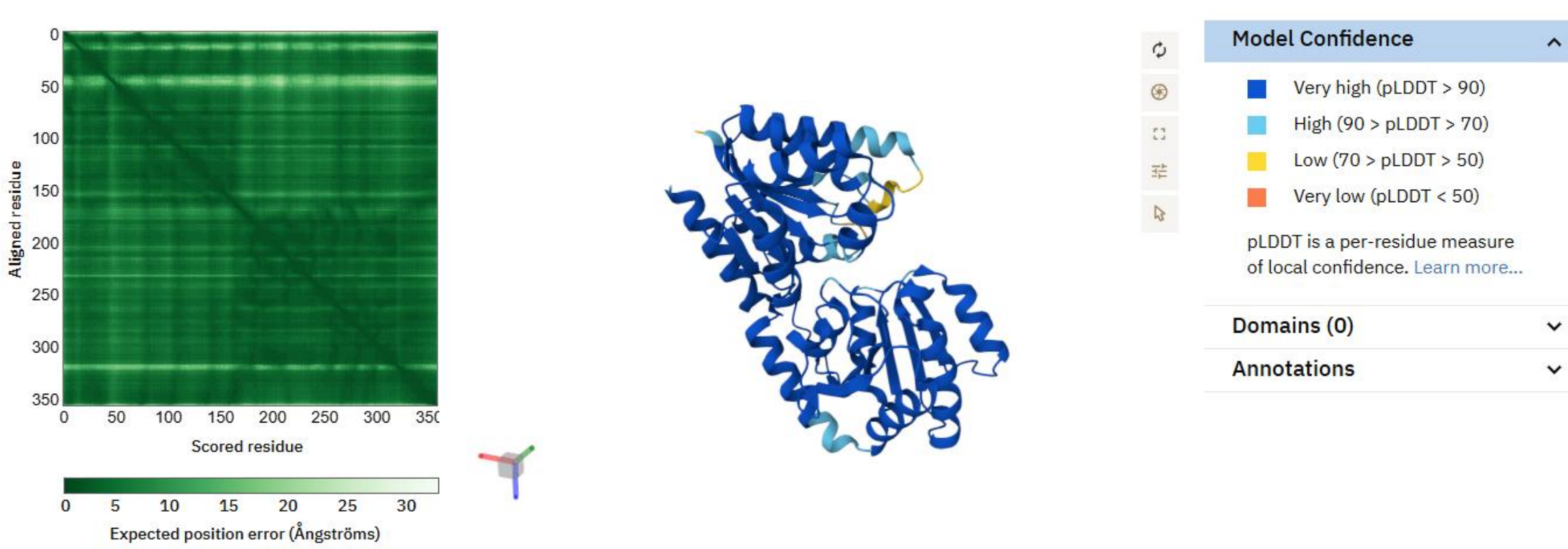

**VpsK**

MRNNRLKRINFLGSPMDVASMSETVVLIRDRILRQEFTQHVVVNVAKLVNMRKDPELAQSVTEC
DIINIDGMGVVWGARFLGHQVPERVAGVDLFLELNAMAAEEQMPVFYLGAKPEIVEKTAAVMQ
AKHPQLVIAGYHHGYFWDDEQAIVEKIRQSGAKLLFVAITSPKKENFINRWQEQLGVLFVMGVG
GTFDVVAGHVSRAPLWMQKAGLEWLYRVLQEPGRMWKRYLITNSRYAWLLLNEKVKPKN

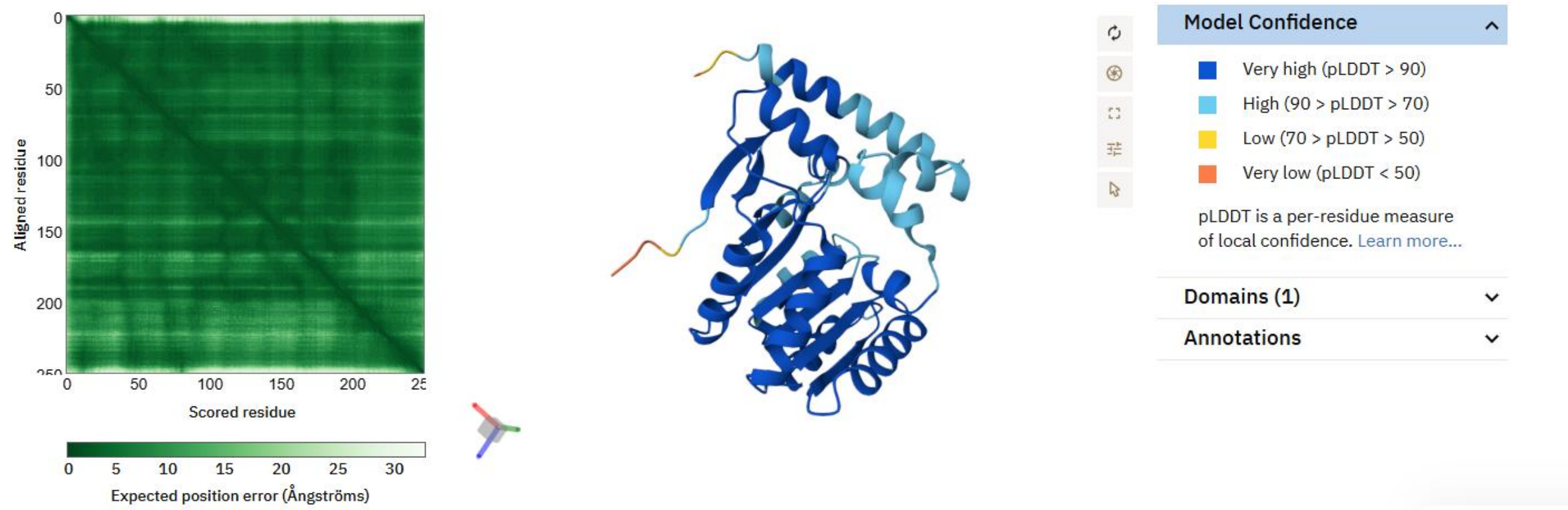


**VpsL**

MMKEKSRIRITNYHGKFFYRLIDSIFILVIMYISIKSNQHVVTMSYISVALLGVLFYSYIAESLDIYS
GWRTAKLRSLSLHTAFCWAATIASLTLLAYFSKTGIEFSRMVMGTWFVGSFVGLIGWRVLAFATIH
YMHKKGLHTKKAVIIGMTTQGQELSNNLLKNPELGIVMQGFYDDRAPERLKEGAPVLGNINDAL
SLAKTGQVQNVYIALPMQAQRRINQILDAFSDSTVNTYIVPDFFTFNLLHSRWYTIGDVNAFSIFD
TPFNGLLNWVKRFEDLVLSSLILLLISPVLLAVAIGVKLSSPGPIIFKQNRYGLDGKPIQVWKFRSM
RVMDNGSHVQQATKGDPRVTRFGAFIRRTSLDELPQFFNVLQGSMSIVGPRPHAVAHNEQYRTIV
NRYMLRHKVKPGITGWAQINGWRGETDTLDKMEKRVQFDLDYIHRWSLWFDLKIVFLTIFKGFV
GKNAY

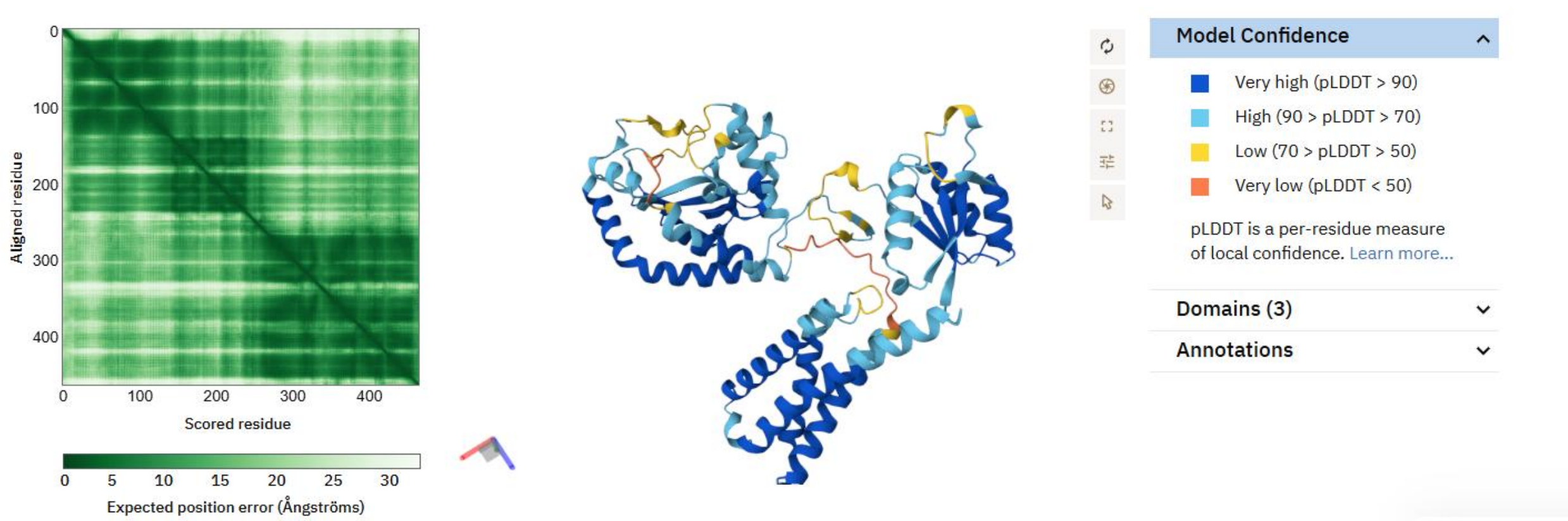

**Supporting Figure 2.** Sequence analysis of PGTs giving full sequence and compared sequence highlighted in yellow. ClustalW generated Multiple sequence alignment is provided below the sequences along with phylogenetic tree.

**VpsL**

MMKEKSRIRITNYHGKFFYRLIDSIFILVIMYISIKSNQHVVTMSYISVALLGVLFYSYIAESLDIYS
GWRTAKLRSLSLHTAFCWAATIASLTLLAYFSKTGIEFSRMVMGAWFVGSFIGLIGWRVLAFATIH
YMHKKGLHTKNAVIIGMTTQGQELSNNLLKNPELGIVMQGFYDDRAPERLKEGAPVLGNINDAL
SLAKTGQVQNVYIALPMQAQRRINQILDAFSDSTVNTYIVPDFFTFNLLHSRWYTIGDVNAFSIFD
TPFNGLLNWVKRFEDLVLSSLILLLISPVLLAVAIGVKLSSPGPIIFKQNRYGLDGKPIQVWKFRSM
RVMDNGSHVQQATKGDPRVTRFGAFIRRTSLDELPQFFNVLQGSMSIVGPRPHAVAHNEQYRTIV
NRYMLRHKVKPGITGWAQINGWRGETDTLDKMEKRVQFDLDYIHRWSLWFDLKIVFLTIFKGFV
GKNAY

**WcaJ *E. coli***

MTNLKKRERAKTNASLISMVQRFSDITIMFAGLWLVCEVSGLSFLYMHLLVALITLVVFQMLGGIT
DFYRSWRGVRAATEFALLLQNWTLSVIFSAGLVAFNNDFDTQLKIWLAWYALTSIGLVVCRSCIRI
GAGWLRNHGYNKRMVAVAGDLAAGQMLMESFRNQPWLGFEVVGVYHDPKPGGVSNDWAGN
LQQLVEDAKAGKIHNVYIAMQMCDGARVKKLVHQLADTTCSVLLIPDVFTFNILHSRLEEMNGV
PVVPLYDTPLSGVNRLLKRAEDIVLATLILLLISPVLCCIALAVKLSSPGPVIFRQTRYGMDGKPIKV
WKFRSMKVMENDKVVTQATQNDPRVTKVGNFLRRTSLDELPQFINVLTGGMSIVGPRPHAVAHN
EQYRQLIEGYMLRHKVKPGITGWAQINGWRGETDTLEKMEKRVEFDLEYIREWSVWFDIKIVFLT
VFKGFVNKAAY

***Cps2E S. pneumoniae***

MNGKTVKSSLAIIQSFLIILLTYLLSAVREAEIVSTTAIALYILHYFVFYISDYGQDFFKRGYLIELVQ
TLKYILFFALAISISNFFLEDRFSISRRGMIYFLTLHALLVYVLNLFIKWYWKRAYLNFKGSKKILLL
TATSRVEKVLDRLIESNEVVGELVAVSVLDKSDFQHDCLKVVAEGEIVNFATHEVVDEVFINLPSE
KYNIGELVSQFETMGIDVTVNLNAFGCSLARNKQIREMAGLNVVTFSTTFYKTSHVIAKRIIDIMG
ALVGLILCGLVSIVLVPLIRKDGGSAIFAQTRIGKNGRQFTFYKFRSMCVDAEAKKRELMEQNTM
QGGMFKVDDDPRITKIGRFIRKTSLDELPQVYNVLKGDMSLVGTRPPTVDEYEHYTPEQKRRLSF
KPGITGLWQVSGRSEIKNFDEVVKLDVAYIDDWTIWKDIEILLKTVKVVFMRDGAK

***PssY C. vibrioides***

MKEQGLPAVNIIACASPLLCTTTSSDCVSVWACELEQPLSDALGKLKDHPVDLGPSTQLLTLRPGG
PLPRVAVDLRKRVLDVVAAALLTALFAPLLLLAALAIKLESPGPALFRQTRGGLGGAPFQILKLRT
MHCREDGPDVAQAQRGDDRVTRVGRILRAASIDELPQLLNVLRGDMSLVGPRPHATAHDDYYSA
RIPEYAARYQARPGLTGLAQVRGLRGGTETVELMRQRIAADIDYIQTWSLWRDLKIVLRTVPSLLT
TDNAY

***WbaP S. enterica***

MDNIDNKYNPQLCKIFLAISDLIFFNLALWFSLGCVYFIFGQVQRFIPQDQLDTRVITHFILSVVCV
GWFWIRLRHYTYRKPFWYELKEIFRTIVIFAIFDLALIAFTKWQFSRYVWVFCWTFALILVPFFRAL
TKHLLNKLGIWKKKTIILGSGQNARGAYSALQSEEMMGFDVIAFFDTDASDVEINMLPVIKDTEN
IWDLNRTGDVHYILAYEYTELEKTHFWLRELSKHHCRSVTVVPSFRGLPLYNTDMSFIFSHEVML
LRIQNNLAKRSSRFLKRTFDIVCSIIILIIASPLMIYLWYKVTRDGGPAIYGHQRVGRHGKPFPCYKF
RSMVMNSQEVLKELLANDPIARAEWEKDFKLKNDPRVTVVGRFIRKTSLDELPQLFNVLKGDMS
LVGPRPIVSDELERYCDDVDYYLMAKPGMTGLWQVSGRNDVDYDTRVYFDSWYVKNWTLWN
DIAILFKTAKVVLQRDGAY

***WecP A. hyrophilia***

MSYQRRHSRWYERVLFSPPSLFFLGAMLAVCLPALERWGWGFWEYFDAVRVNTLGGAFVAFLLT
GIVLYRFLRYPGASPVAYMIPTVTTLYGSLVGALFFLRLPYSRQVLFESYVVALLCCWVVVYFIGRR
YRTPKYALLPFGDYQPLMHHTCVEWRLLDKPDLGAVRYDAVVADLRDDDLAGEWERFLARCAL
AHIPVYHIKQISETLTGRVKIDHLHENQLGSLLPSPIYAFIKRGMDILAAVIAIPLFSPLMLATAVLIKL
ESPGPVMFLQNRVGKGNRDFRIYKFRSMCQNSEQHGAQFAQDGDMRVTRVGKVIRKLRIDELPQ
FFNVLKGDMSLIGPRPEQRTFVDQFDREIPFYMYRHIVRPGISGWAQVVHGYAADADDTRIKIEH
DFYYIKNFSLWLDVLIVFKTIRTILTGFGAR

```
 CLUSTAL 2.1 Multiple Sequence Alignments


Sequence type explicitly set to Protein
Sequence format is Pearson
Sequence 1: VpsL         190 aa
Sequence 2: WcaJ         202 aa
Sequence 3: Cps2E        200 aa
Sequence 4: PssY         202 aa
Sequence 5: WbaP         206 aa
Sequence 6: WecP         199 aa
Start of Pairwise alignments
Aligning...

Sequences (1:2) Aligned. Score:  76
Sequences (1:3) Aligned. Score:  34
Sequences (1:4) Aligned. Score:  48
Sequences (1:5) Aligned. Score:  35
Sequences (1:6) Aligned. Score:  42

CLUSTAL 2.1 multiple sequence alignment


VpsL            ------------RFEDLVLSSLILLLISPVLLAVAIGVKLSSPGPIIFKQNRYGLDGKPI
WcaJ            DTPLSGVNRLLKRAEDIVLATLILLLISPVLCCIALAVKLSSPGPVIFRQTRYGMDGKPI
PssY            GPLPRVAVDLRKRVLDVVAAALLTALFAPLLLLAALAIKLESPGPALFRQTRGGLGGAPF
WecP            -LLPSPIYAFIKRGMDILAAVIAIPLFSPLMLATAVLIKLESPGPVMFLQNRVGKGNRDF
Cps2E           -----TSHVIAKRIIDIMGALVGLILCGLVSIVLVPLIRKDG-GSAIFAQTRIGKNGRQF
WbaP            --LAKRSSRFLKRTFDIVCSIIILIIASPLMIYLWYKVTRDG-GPAIYGHQRVGRHGKPF
                            *  *:: : :   : . :       :  .. *. :: : * *  .  :

VpsL            QVWKFRSMRVMDNGSHVQQATKG---------------DPRVTRFGAFIRRTSLDELPQF
WcaJ            KVWKFRSMKVMENDKVVTQATQN---------------DPRVTKVGNFLRRTSLDELPQF
PssY            QILKLRTMHCREDGPDVAQAQRG---------------DDRVTRVGRILRAASIDELPQL
WecP            RIYKFRSMCQNSEQHGAQFAQDG---------------DMRVTRVGKVIRKLRIDELPQF
Cps2E           TFYKFRSMCVDAEAKKRELMEQN----TMQGGMFKVDDDPRITKIGRFIRKTSLDELPQV
WbaP            PCYKFRSMVMNSQEVLKELLANDPIARAEWEKDFKLKNDPRVTVVGRFIRKTSLDELPQL
                   *:*:*    :         .               * *:* .* .:*   :*****.

VpsL            FNVLQGSMSIVGPRPHAVAHNEQYRTIVNRYMLRHKVKPGITGWAQINGWRGETDTLDKM
WcaJ            INVLTGGMSIVGPRPHAVAHNEQYRQLIEGYMLRHKVKPGITGWAQINGWRGETDTLEKM
PssY            LNVLRGDMSLVGPRPHATAHDDYYSARIPEYAARYQARPGLTGLAQVRGLRGGTETVELM
WecP            FNVLKGDMSLIGPRPEQRTFVDQFDREIPFYMYRHIVRPGISGWAQVVHG--YAADADDT
Cps2E           YNVLKGDMSLVGTRP---PTVDEYEHYTPEQKRRLSFKPGITGLWQVSGR----SEIKNF
WbaP            FNVLKGDMSLVGPRP---IVSDELERYCDDVDYYLMAKPGMTGLWQVSGR----NDVD-Y
                 *** *.**::*.**      :               :**::*  *:          .

VpsL            EKRVQFDLDYIHRWSLWFDLKIVFLTIFKGFVGKNAY
WcaJ            EKRVEFDLEYIREWSVWFDIKIVFLTVFKGFVNKAAY
PssY            RQRIAADIDYIQTWSLWRDLKIVLRTVPSLLTTDNAY
WecP            RIKIEHDFYYIKNFSLWLDVLIVFKTIRTILTGFGAR
Cps2E           DEVVKLDVAYIDDWTIWKDIEILLKTVKVVFMRDGAK
WbaP            DTRVYFDSWYVKNWTLWNDIAILFKTAKVVLQRDGAY
                   :  *  *:  :::* *: *:: *    :    *
```

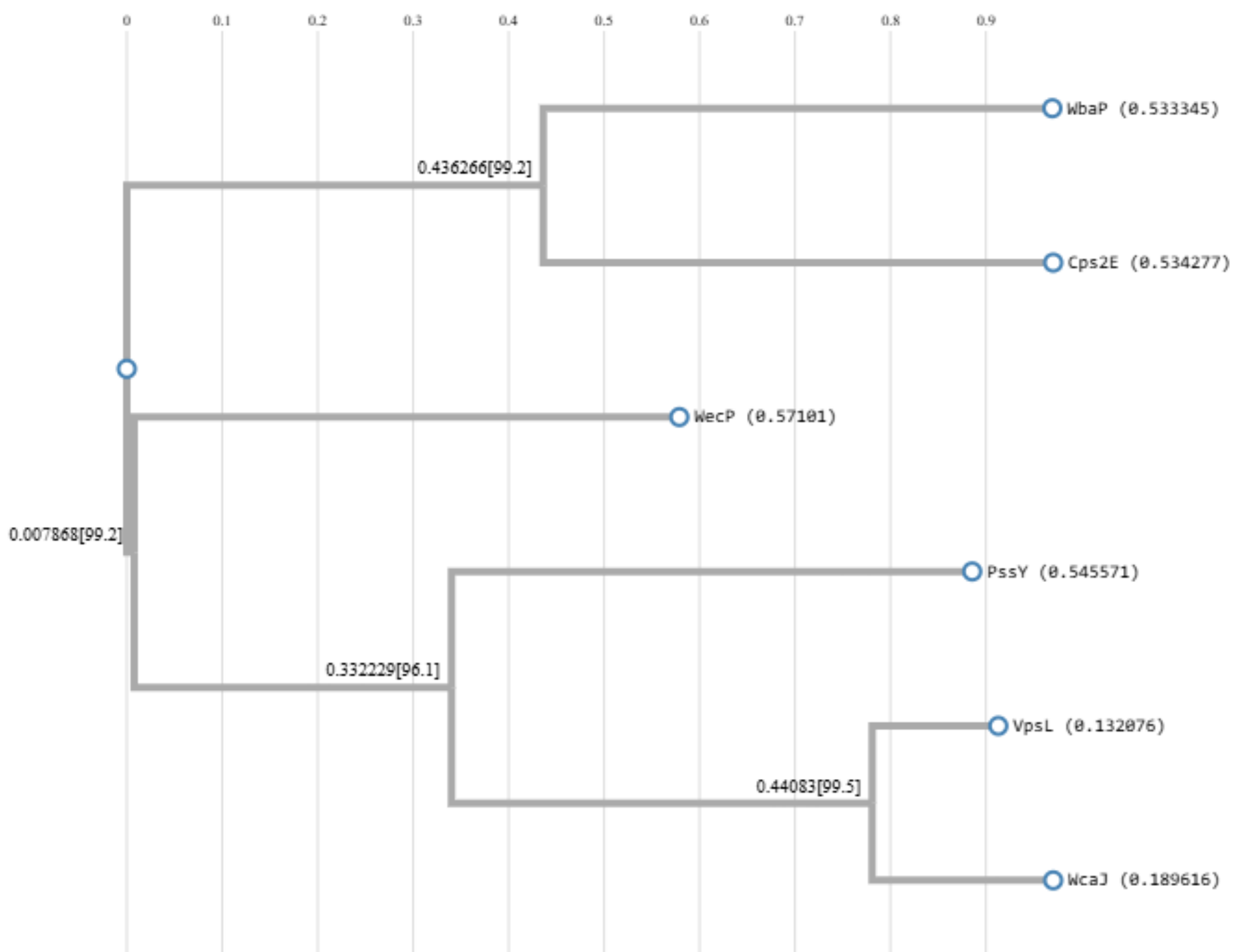
0
0.1
0.2
0.3
0.4
0.5
0.6
0.7
0.8
0.9
WbaP (0.533345)
0.436266[99.2]
Cps2E (0.534277)
WecP (0.57101)
0.007868[99.2]
PssY (0.545571)
0.332229[96.1]
VpsL (0.132076)
0.44083[99.5]
WcaJ (0.189616)

**Supporting Figure 3.** Predicted structure comparisons for each PGT pair. Colors are based on RMSD in angstroms difference between structures.

**VpsL to WcaJ**

RMSD between 190 pruned atom pairs is 0.357 angstroms; (across all 190 pairs: 0.357)

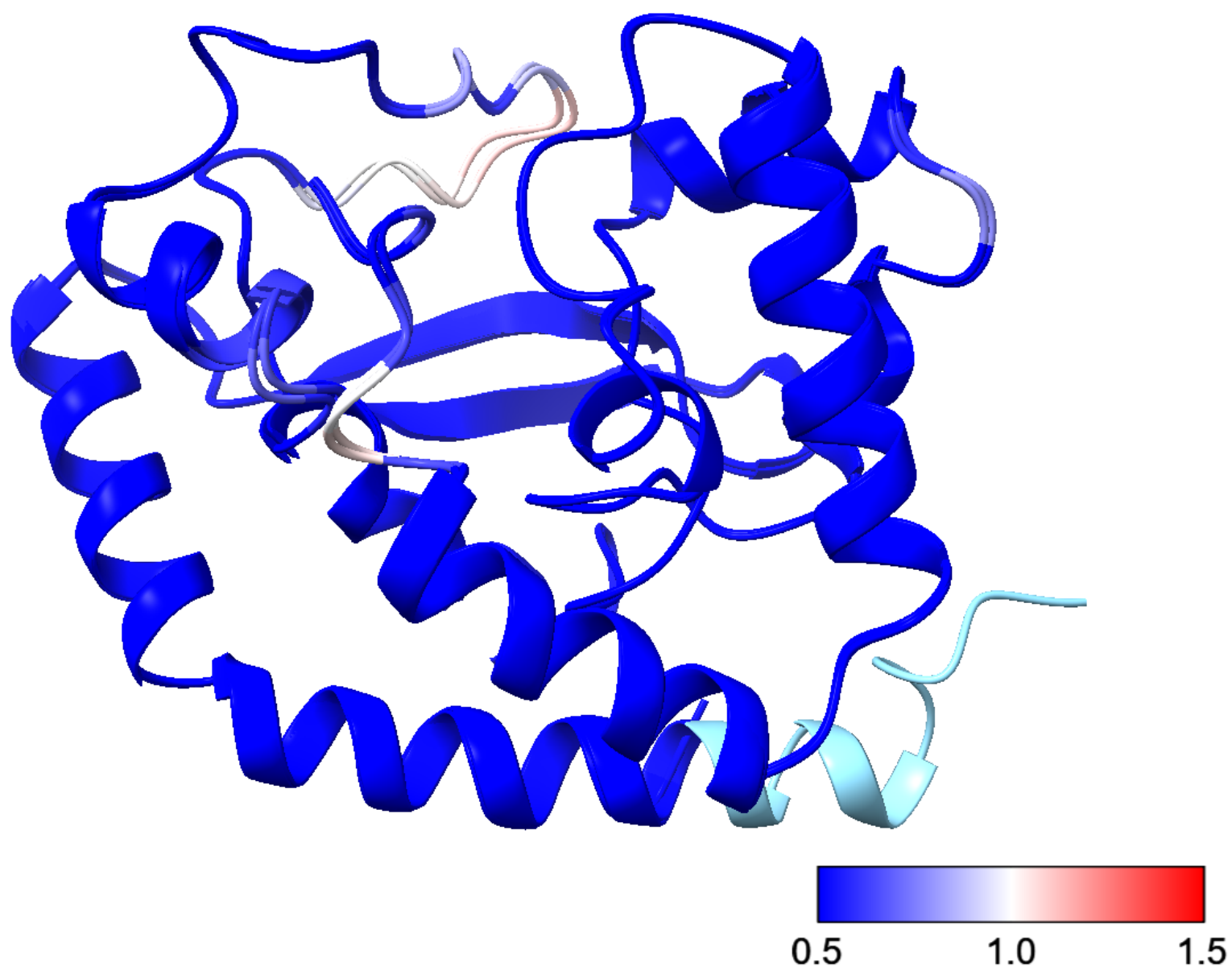


**VpsL to Cps2E**

RMSD between 117 pruned atom pairs is 1.025 angstroms; (across all 182 pairs: 4.239)

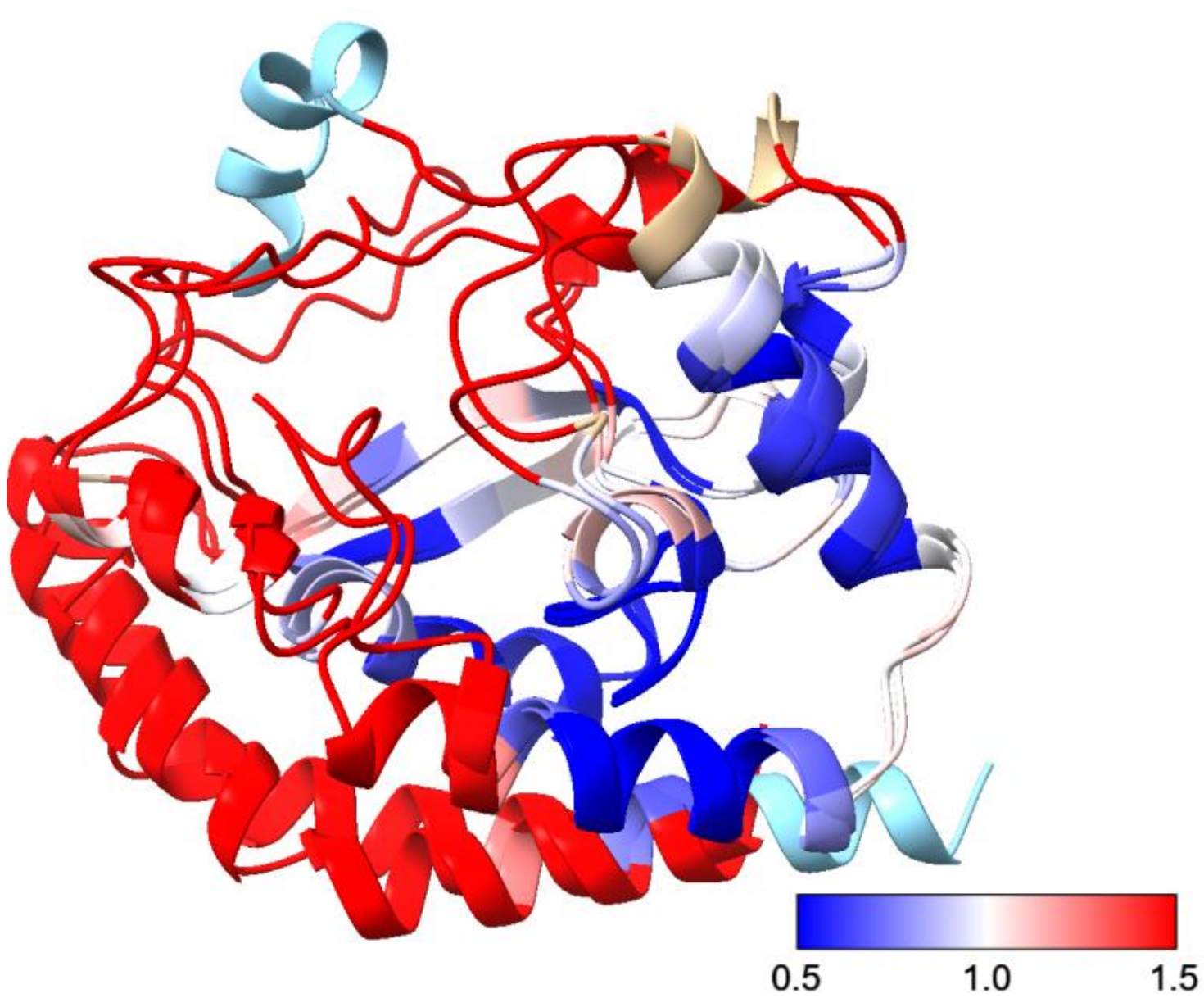

**VpsL to PssY**

RMSD between 185 pruned atom pairs is 0.955 angstroms; (across all 190 pairs: 1.080)

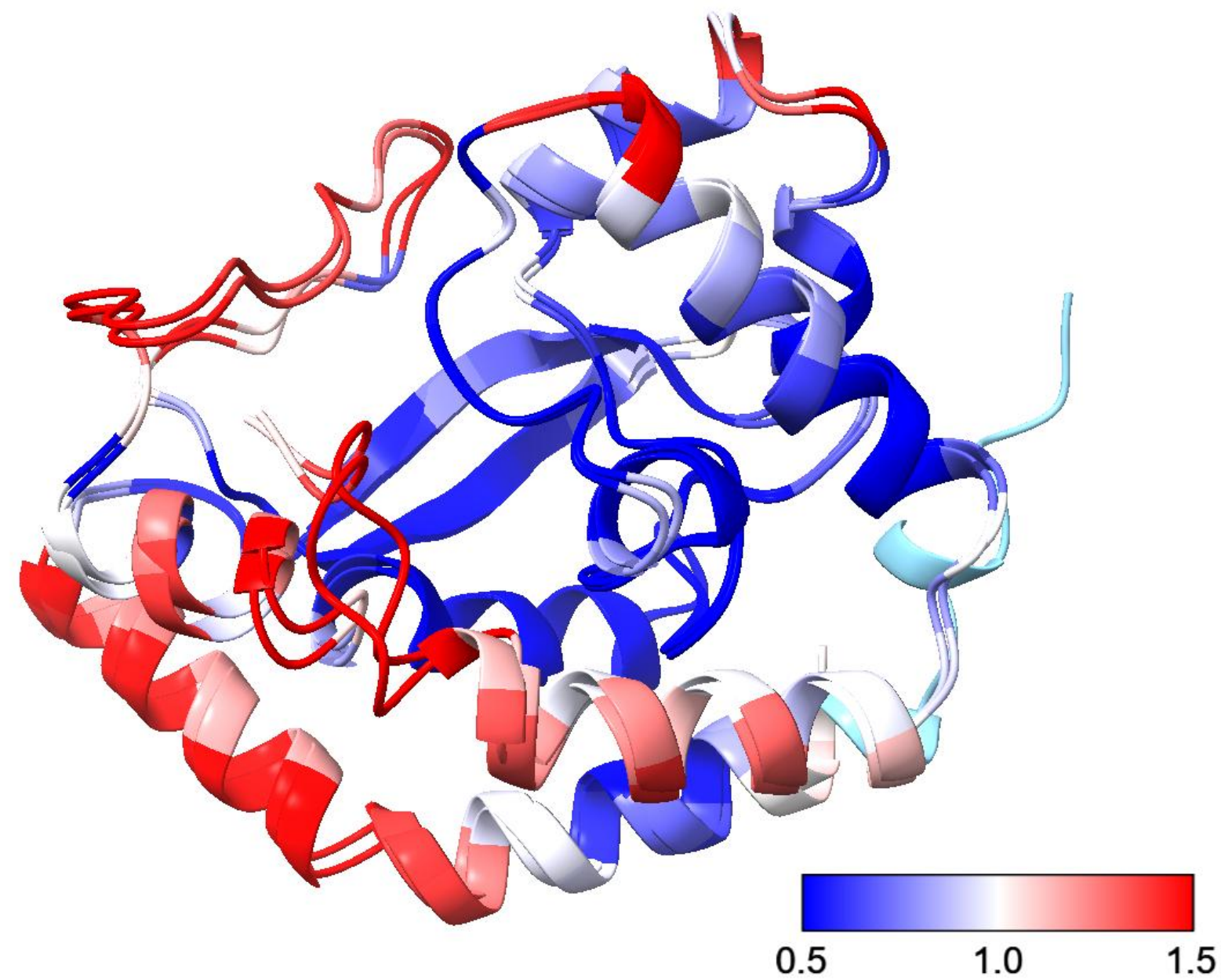


**VpsL to WbaP**

RMSD between 139 pruned atom pairs is 0.758 angstroms; (across all 179 pairs: 3.468)

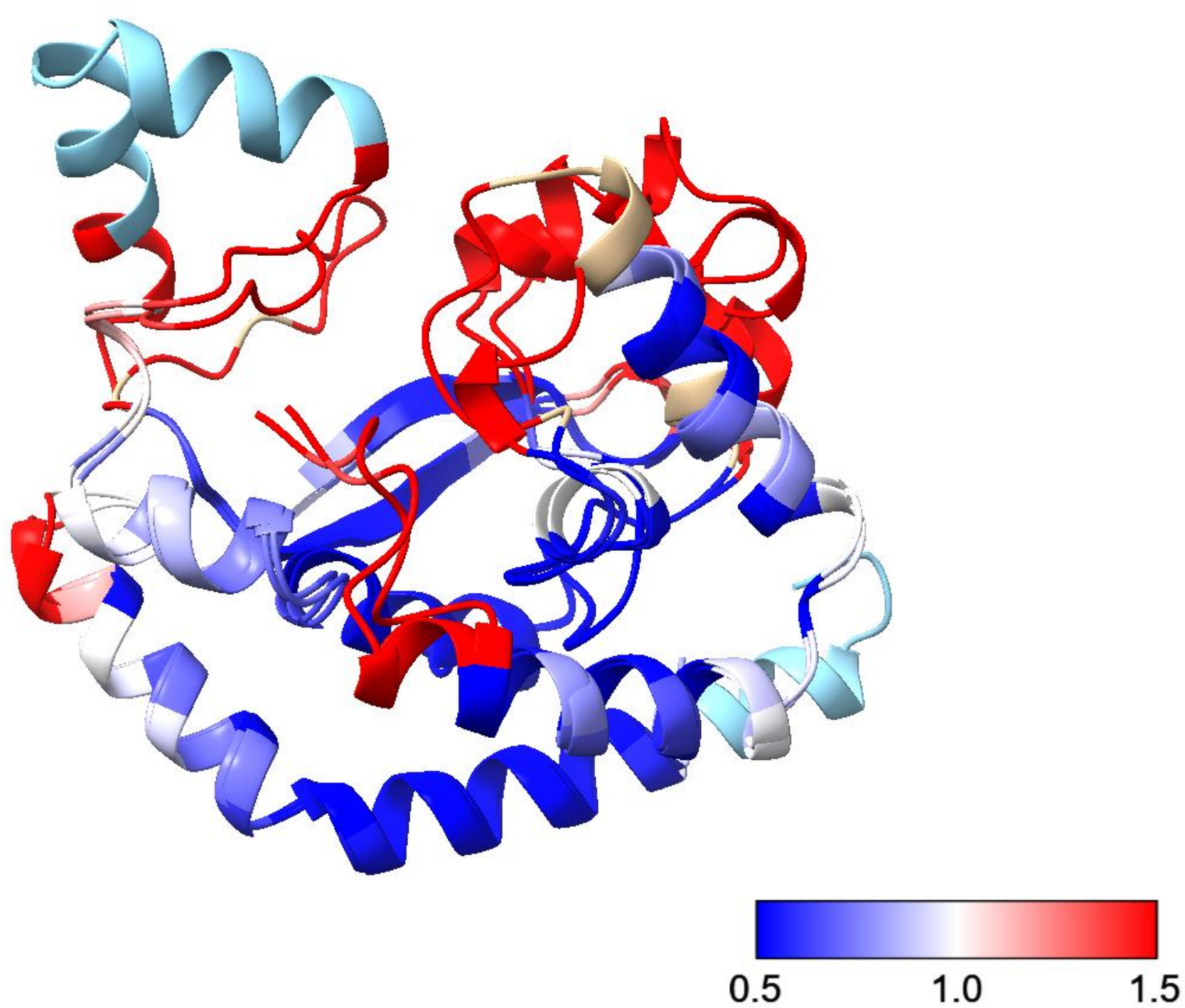

**VpsL to WecP**

**RMSD between 143 pruned atom pairs is 0.993 angstroms; (across all 184 pairs: 2.244)**

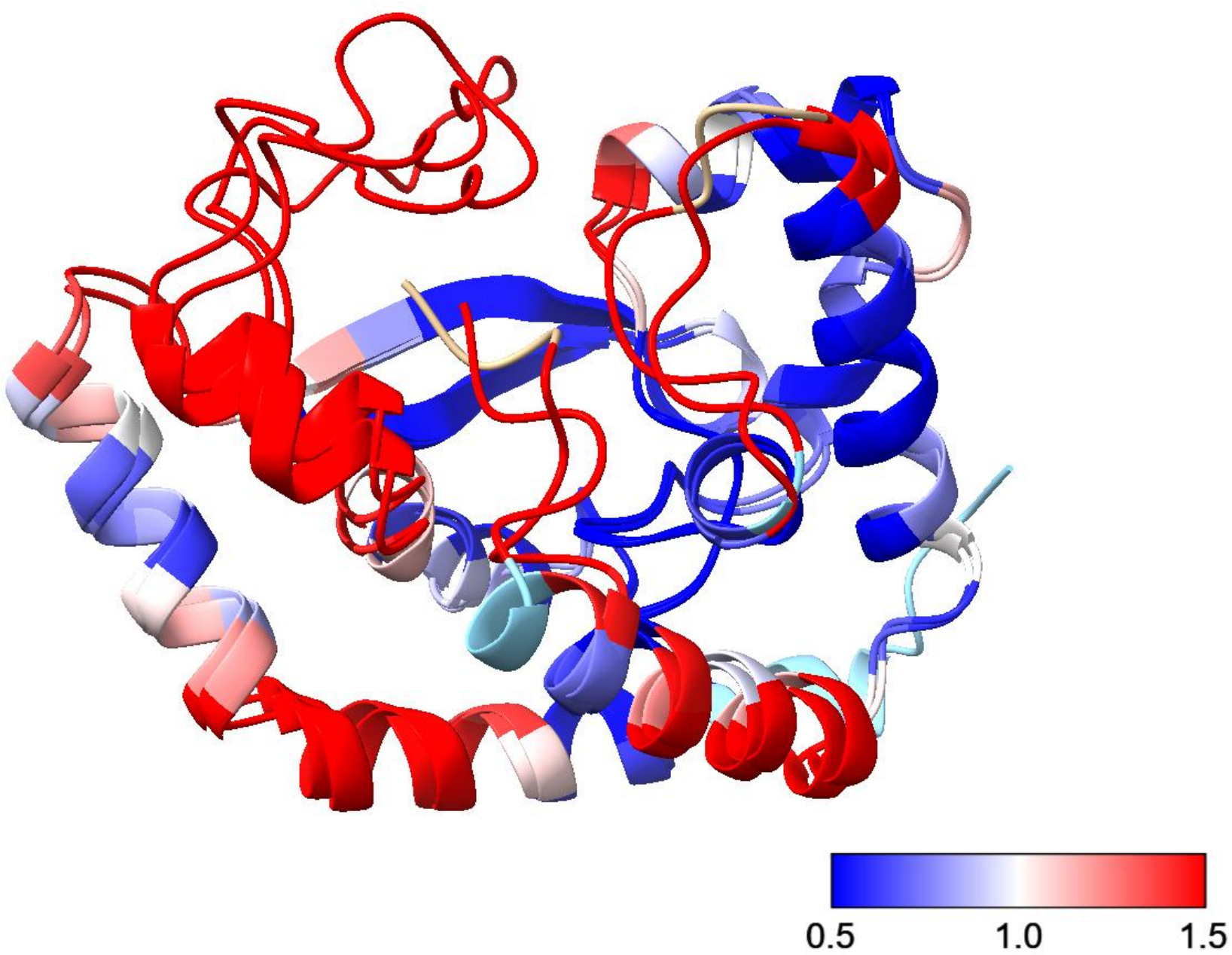

**Supporting Figure 4. Wec/Vps protein sequence comparisons.**

**WecG (Uniprot ID: P27836)**

MNNNTTAPTYTLRGLQLIGWRDMQHALDYLFADGQLKQGTLVAINAEKMLTIEDNAEVRELINA
AEFKYADGISVVRSVRKKYPQAQVSRVAGADLWEELMARAGKEGTPVFLVGGKPEVLAQTEAK
LRNQWNVNIVGSQDGYFKPEQRQALFERIHASGAQIVTVAMGSPKQEIIMRDCRLVHPDALYMG
VGGTYDVFTGHVKRAPKIWQTLGLEWLYRLLSQPSRIKRQLRLLRYLRWHYTGNL

**VpsK (Uniprot ID: A0A2H4NFE3)**

MRNNRLKRINFLGSPMDVASMSETVVLIRDRILRQEFTQHVVVNVAKLVNMRKDPELAQSVTEC
DIINIDGMGVVWGARFLGHQVPERVAGVDLFLELNAMAAEEQMPVFYLGAKPEIVEKTAAVMQ
AKHPQLVIAGYHHGYFWDDEQAIVEKIRQSGAKLLFVAITSPKKENFINRWQEQLGVLFVMGVG
GTFDVVAGHVSRAPLWMQKAGLEWLYRVLQEPGRMWKRYLITNSRYAWLLLNEKVKPKN

Alignment Score 326: pBLAST alignment 39% identical, 57% similar 2% gaps

```
CLUSTAL 2.1 multiple sequence alignment


WecG            -MNNNTTAPTYTLRGLQLIGWRDMQHALDYLFADGQLKQGTLVAINAEKMLTIEDNAEVR
VpsK            MRNNRLKRINFLGSPMDVASMSETVVLIRDRILRQEFTQ--HVVVNVAKLVNMRKDPELA
                  **. .  .:    ::: .  :    :   :   ::.*   *.:*. *::.:..:.*:

WecG            ELINAAEFKYADGISVVRSVRKKYPQAQVSRVAGADLWEELMARAGKEGTPVFLVGGKPE
VpsK            QSVTECDIINIDGMGVVWGARFLG-HQVPERVAGVDLFLELNAMAAEEQMPVFYLGAKPE
                : :. .::   **:.** ..*    :   .****.**: ** * *.:*  *** :*.***

WecG            VLAQTEAKLRNQWNVNIVGSQDGYFKPEQRQALFERIHASGAQIVTVAMGSPKQEIIMRD
VpsK            IVEKTAAVMQAKHPQLVIAGYHHGYFWDDEQAIVEKIRQSGAKLLFVAITSPKKENFINR
                :: :* * :: :    ::.. .  :  ::.**:.*:*: ***::: **: ***:* ::.

WecG            CRLVHPDALYMGVGGTYDVFTGHVKRAPKIWQTLGLEWLYRLLSQPSRIKRQLRLLRYLR
VpsK            WQEQLGVLFVMGVGGTFDVVAGHVSRAPLWMQKAGLEWLYRVLQEPGRMWKRYLITNSRY
                 :      : ******:**.:***.***   *. *******:*.:*.*: ::  : .

WecG            WHYTGNL------
VpsK            AWLLLNEKVKPKN
                     *
```

**WecB (Uniprot ID: P27828)**

MKVLTVFGTRPEAIKMAPLVHALAKDPFFEAKVCVTAQHREMLDQVLKLFSIVPDYDLNIMQPG
QGLTEITCRILEGLKPILAEFKPDVVLVHGDTTTTLATSLAAFYQRIPVGHVEAGLRTGDLYSPWPE
EANRTLTGHLAMYHFSPTETSRQNLLRENVADSRIFITGNTVIDALLWVRDQVMSSDKLRSELAA
NYPFIDPDKKMILVTGHRRESFGRGFEEICHALADIATTHQDIQIVYPVHLNPNVREPVNRILGHV
KNVILIDPQEYLPFVWLMNHAWLILTDSGGIQEEAPSLGKPVLVMRDTTERPEAVTAGTVRLVGT
DKQRIVEEVTRLLKDENEYQAMSRAHNPYGDGQACSRILEALKNNRISL

**VpsA (Uniprot ID: A0A0E4CCM6)**

MKKVLIVFGTRPEAIKMAPLVQQLCQDNRFVAKVCVTGQHREMLDQVLELFSITPDFDLNIMEPG
QTLNGVTSKILLGMQQVLSSEQPDVVLVHGDTATTFAASLAAYYQQIPVGHVEAGLRTGNIYSPW
PEEGNRKLTAALTQYHFAPTDTSRANLLQENYNAENIFVTGNTVIDALLAVREKIHTDMDLQATL
ESQFPMLDASKKLILVTGHRRESFGGGFERICQALITTAEQHPECQILYPVHLNPNVREPVNKLLK
GVSNIVLIEPQQYLPFVYLMDRAHIILTDSGGIQEEAPSLGKPVLVMRETTERPEAVAAGTVKLVG
TNQQQICDALSLLLTDPQAYQAMSQAHNPYGDGKACQRIADILAK

Alignment Score 1558: pBLAST alignment 67% identical, 81% similar, 0% gaps

```
CLUSTAL 2.1 multiple sequence alignment


WecB            -MKVLTVFGTRPEAIKMAPLVHALAKDPFFEAKVCVTAQHREMLDQVLKLFSIVPDYDLN
VpsA            MKKVLIVFGTRPEAIKMAPLVQQLCQDNRFVAKVCVTGQHREMLDQVLELFSITPDFDLN
                  *** ***************: *.:*  * ******.**********:****.**:***

WecB            IMQPGQGLTEITCRILEGLKPILAEFKPDVVLVHGDTTTTLATSLAAFYQRIPVGHVEAG
VpsA            IMEPGQTLNGVTSKILLGMQQVLSSEQPDVVLVHGDTATTFAASLAAYYQQIPVGHVEAG
                **:*** *. :*.:** *:: :*:. :**********:**:*:****:**:*********

WecB            LRTGDLYSPWPEEANRTLTGHLAMYHFSPTETSRQNLLRENVADSRIFITGNTVIDALLW
VpsA            LRTGNIYSPWPEEGNRKLTAALTQYHFAPTDTSRANLLQENYNAENIFVTGNTVIDALLA
                ****::*******.**.**. *: ***:**:*** ***:**   ..**:**********

WecB            VRDQVMSSDKLRSELAANYPFIDPDKKMILVTGHRRESFGRGFEEICHALADIATTHQDI
VpsA            VREKIHTDMDLQATLESQFPMLDASKKLILVTGHRRESFGGGFERICQALITTAEQHPEC
                **::: :. .*:: * :::*::*..**:************ ***.**:**   *  * :

WecB            QIVYPVHLNPNVREPVNRILGHVKNVILIDPQEYLPFVWLMNHAWLILTDSGGIQEEAPS
VpsA            QILYPVHLNPNVREPVNKLLKGVSNIVLIEPQQYLPFVYLMDRAHIILTDSGGIQEEAPS
                **:**************::*  *.*::**:**:*****:**::* :**************

WecB            LGKPVLVMRDTTERPEAVTAGTVRLVGTDKQRIVEEVTRLLKDENEYQAMSRAHNPYGDG
VpsA            LGKPVLVMRETTERPEAVAAGTVKLVGTNQQQICDALSLLLTDPQAYQAMSQAHNPYGDG
                *********:********:****:****::*:* : :: **.* : *****:********

WecB            QACSRILEALKNNRISL
VpsA            KACQRIADILAK-----
                :**.** : * :
```

**WecC (Uniprot ID: P27829)**

MSFATISVIGLGYIGLPTAAAFASRQKQVIGVDINQHAVDTINRGEIHIVEPDLASVVKTAVEGGFL
RASTTPVEADAWLIAVPTPFKGDHEPDMTYVESAARSIAPVLKKGALVILESTSPVGSTEKMAEW
LAEMRPDLTFPQQVGEQADVNIAYCPERVLPGQVMVELIKNDRVIGGMTPVCSARASELYKIFLE
GECVVTNSRTAEMCKLTENSFRDVNIAFANELSLICADQGINVWELIRLANRHPRVNILQPGPGVG
GHCIAVDPWFIVAQNPQQARLIRTAREVNDHKPFWVIDQVKAAVADCLAATDKRASELKIACFGL
AFKPNIDDLRESPAMEIAELIAQWHSGETLVVEPNIHQLPKKLTGLCTLAQLDEALATADVLVMLV
DHSQFKVINGDNVHQQYVVDAKGVWR

**VpsB (Uniprot ID: A0A0H6YTR0)**

MFNKVSVIGLGYIGLPTAAVLASRGLDVVGVDVNAHAVDTINKGEIHIVEPDLDIVVRSAVMTGK
LKATLTAEPADAFLVAVPTPFKGDNHEPDLSYIEAAAKTIAPVLAKGNLVVLESTSPVGTTEKLAQ
WLAEARPDLTFPQQKGIAADVKIAYCPERVLPGYVLQELVSNDRVIGGVSKACSEKAVALYKTFV
KGECIVTNARTAEMAKLTENSFRDVNIAFANELSLVCNKLKINVWELIRLANRHPRVSILNPGPGV
GGHCIAVDPWFIVNSCPDEAQLIRTAREVNDGKPHWVVDQVKQAADQFKKPIIACLGLAFKADID
DLRESPALDITKHLAESKVGQLLAVEPNITELPSKLAKQGIELVSLERALELANIVVVLVDHKEFKA
ADKTEFAKKVVIDTRGVVA

Alignment Score 1667 pBLAST alignment 65% identical, 77% similar, 3% gaps

```
CLUSTAL 2.1 multiple sequence alignment


WecC            MSFATISVIGLGYIGLPTAAAFASRQKQVIGVDINQHAVDTINRGEIHIVEPDLASVVKT
VpsB            -MFNKVSVIGLGYIGLPTAAVLASRGLDVVGVDVNAHAVDTINKGEIHIVEPDLDIVVRS
                  * .:**************.:***  :*:***:* *******:**********  **::

WecC            AVEGGFLRASTTPVEADAWLIAVPTPFKG-DHEPDMTYVESAARSIAPVLKKGALVILES
VpsB            AVMTGKLKATLTAEPADAFLVAVPTPFKGDNHEPDLSYIEAAAKTIAPVLAKGNLVVLES
                **  * *:*: *.  ***:*:******** :****::*:*:**::***** ** **:***

WecC            TSPVGSTEKMAEWLAEMRPDLTFPQQVGEQADVNIAYCPERVLPGQVMVELIKNDRVIGG
VpsB            TSPVGTTEKLAQWLAEARPDLTFPQQKGIAADVKIAYCPERVLPGYVLQELVSNDRVIGG
                *****:***:*:**** ********* *  ***:*********** *: **:.*******

WecC            MTPVCSARASELYKIFLEGECVVTNSRTAEMCKLTENSFRDVNIAFANELSLICADQGIN
VpsB            VSKACSEKAVALYKTFVKGECIVTNARTAEMAKLTENSFRDVNIAFANELSLVCNKLKIN
                :: .** :*  *** *::***:***:*****.********************:* .  **

WecC            VWELIRLANRHPRVNILQPGPGVGGHCIAVDPWFIVAQNPQQARLIRTAREVNDHKPFWV
VpsB            VWELIRLANRHPRVSILNPGPGVGGHCIAVDPWFIVNSCPDEAQLIRTAREVNDGKPHWV
                **************.**:****************** . *::*:********** **.**

WecC            IDQVKAAVADCLAATDKRASELKIACFGLAFKPNIDDLRESPAMEIAELIAQWHSGETLV
VpsB            VDQVKQAADQFKKP--------IIACLGLAFKADIDDLRESPALDITKHLAESKVGQLLA
                :**** *. :   .         ***:*****.:*********::*:: :*: : *: *.

WecC            VEPNIHQLPKKLTG-LCTLAQLDEALATADVLVMLVDHSQFKVINGDNVHQQYVVDAKGV
VpsB            VEPNITELPSKLAKQGIELVSLERALELANIVVVLVDHKEFKAADKTEFAKKVVIDTRGV
                ***** :**.**:     *..*:.**  *:::*:****.:**. :  :. :: *:*::**

WecC            WR
VpsB            VA
```

**Supporting Figure 5. VpsJ protein sequence and structure comparison**

**VpsJ (Uniprot ID:A0A2H4NFC8)**

MTINGICTAILRKARTEQFAGYDPFDGLNSSWFKRLPISKDSIFGLAWIQLFKRSPINLRPWLGVPK
ARNPKGVALFILGLIEEYQVTKDPALLTEAEQLAQWLVTQRCDEQIWHGACWGYHFDWKARAF
FVPKGKPNVITTVYVSRALHQLGQLTGRADWVDLALRSGDFIAGHLLTQNESGEFFAYIPGETAF
VHNASLWAAAWLAVVGRETSRPDYTELALRIARNSAQAQAADGSWAYGTRHHHGFIDGFHTGY
NLEALHLIRTTLAINEFDEVIARGFHFYKQHLFEADGTAKYYHNNPYPLDPHSVSQAIITLLSVGG
EESDVVLVKRVIQRAIQTLYMPERDQFVYQRNKRWVNRIHYARWTQAWMYFSFRFYLRVTNEE

**Human GlcNAc 2-epimerase (Uniprot ID: P51606)**
MSKGLPARQDMEKERETLQAWKERVGQELDRVVAFWMEHSHDQEHGGFFTCLGREGRVYDDL
KYVWLQGRQVWMYCRLYRTFERFRHAQLLDAAKAGGEFLLRYARVAPPGKKCAFVLTRDGRPV
KVQRTIFSECFYTMAMNELWRATGEVRYQTEAVEMMDQIVHWVQEDASGLGRPQLQGAPAAEP
MAVPMMLLNLVEQLGEADEELAGKYAELGDWCARRILQHVQRDGQAVLENVSEGGKELPGCL
GRQQNPGHTLEAGWFLLRHCIRKGDPELRAHVIDKFLLLPFHSGWDPDHGGLFYFQDADNFCPT
QLEWAMKLWWPHSEAMIAFLMGYSDSGDPVLLRLFYQVAEYTFRQFRDPEYGEWFGYLSREGK
VALSIKGGPFKGCFHVPRCLAMCEEMLGALLSRPAPAPSPAPTPACRGAE

**No sequence similarity found**

**VpsJ structure comparison with homo sapiens GlcNAc-2-epimerase (P51606)**

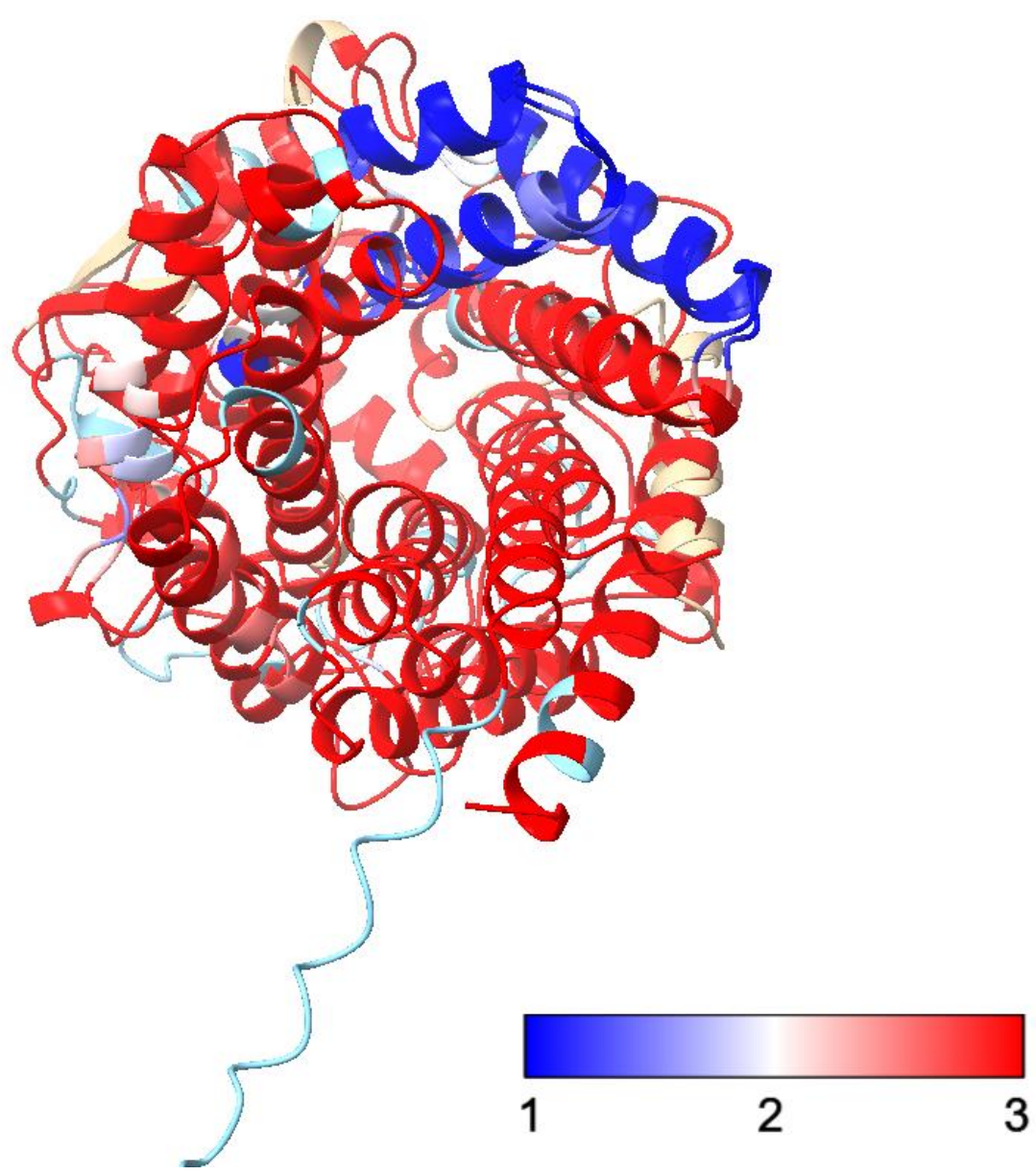

**Supporting Figure 6. VpsH sequence and structure comparison**

**VpsH A0A0H7AHV2**

MFTSSLYVKAPIWLQNVMLSVRALSRQMVRENGEMRAVLQEIQANEFSRTHLADYQHKQLKKT
LENARNHVPFYQDLEVPNLELEAFPYLDKSVLRRDHKQFISQNKPSVVVKGATSGTTGTPLTILQ
DRHSVIREQAFVARQLAWAGYRKGDKRAWIRGDMVVPLSQKQGPFWRYSWFEQMIVLSSFHLT
PKTMSSYLQAMVDFGVEVIQAYPSSIATLARYLEANQSYYPAKLKSIMTSSESLSAEDRQVIETRF
GCKVFDWYGLFERVAAIGQCEHGRYHLLSDYSHVELLAAGDGRYELVGTNFNNQYFPLIRYKTG
DHVHLSENEACPCGRVFPVIERIEGRIGDYLVGEDGQKVHILNHIPKGIAGIMACQFVQSERGQIV
VKVVADAEQFNQEQQKQLISNTQARLGSSLNVTIDIVSALERTANGKIRQAICTIKDVQ

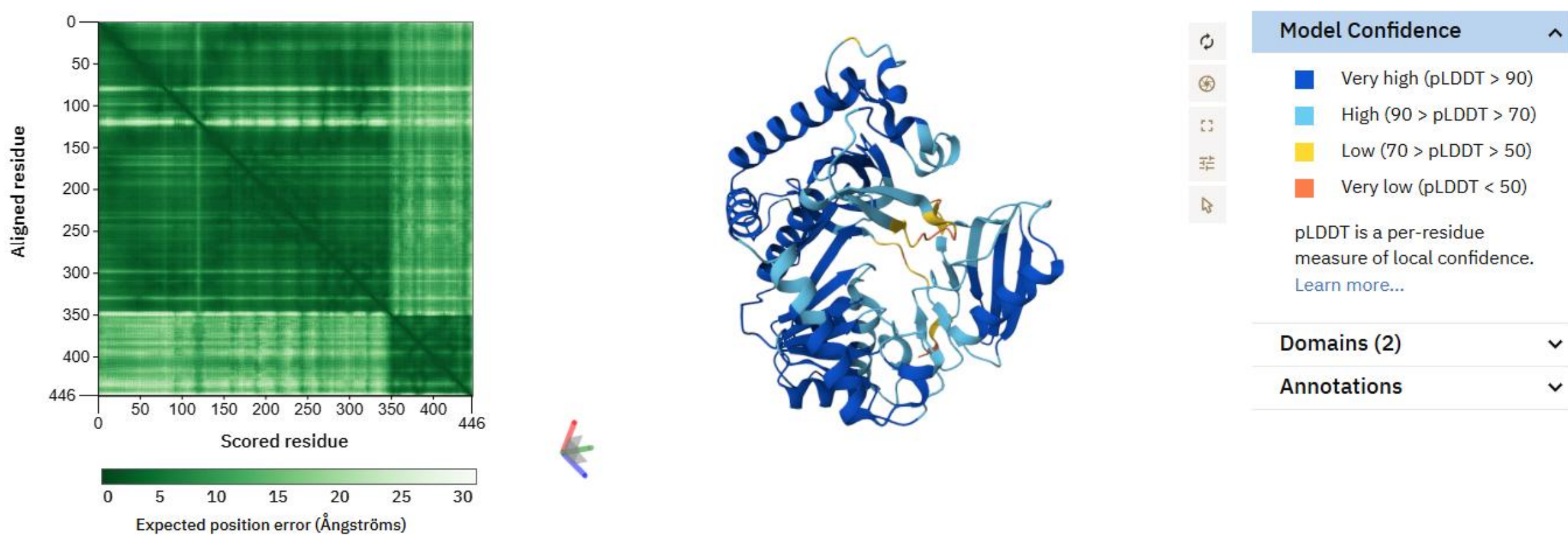


**Phenylacetate CoA Ligase (E. coli) (Uniprot ID: P76085)**

MITNTKLDPIETASVDELQALQTQRLKWTLKHAYENVPMYRRKFDAAGVHPDDFRELSDL
RKFPCTTKQDLRDNYPFDTFAVPMEQVVRIHASSGTTGKPTVVGYTQNDIDNWANIVARS
LRAAGGSPKDKIHVAYGYGLFTGGLGAHYGAERLGATVIPMSGGQTEKQAQLIRDFQPDM
IMVTPSYCLNLIEELERQLGGDASGCSLRVGVFGAEPWTQAMRKEIERRLGITALDIYGL
SEVMGPGVAMECLETTDGPTIWEDHFYPEIVNPHDGTPLADGEHGELLFTTLTKEALPVI
RYRTRDLTRLLPGTARTMRRMDRISGRSDDMLIIRGVNVFPSQLEEEIVKFEHLSPHYQL
EVNRRGHLDSLSVKVELKESSLTLTHEQRCQVCHQLRHRIKSMVGISTDVMIVNCGSIPR
SEGKACRVFDLRNIVGA

| Score | Expect | Method | Identities | Positives | Gaps |
|---|---|---|---|---|---|
| 57.0 bits(136) | 4e-13 | Compositional matrix adjust. | 89/330(27%) | 132/330(40%) | 42/330(12%) |

```
Query  52   LADYQHKQLKKTLENARNHVPFYQ-DLEVPNL---------ELEAFPYLDKSVLRRDHKQ  101
            L   Q ++LK TL++A  +VP Y+   +   +         +L  FP   K  LR D+
Sbjct  18   LQALQTQRLKWTLKHAYENVPMYRRKFDAAGVHPDDFRELSDLRKFPCTTKQDLR-DNYP  76

Query  102  FISQNKP--SVVVKGATSGTTGTPLTILQDRHSVIREQAFVARQLAWAGYRKGDKRAWIR  159
            F +   P   VV   A+SGTTG P  +   ++ +      VAR L  AG    DK
Sbjct  77   FDTFAVPMEQVVRIHASSGTTGKPTVVGYTQNDIDNWANIVARSLRAAGGSPKDKIHVAY  136

Query  160  GDMVVPLSQKQGPFWRYSWFEQMIVLSSFHLTPKTMSSYLQAMVDFGVEVIQAYPSS---  216
            G  +   +   G  +        ++  S   T K      Q + DF  ++I   PS
Sbjct  137  GYGL--FTGGLGAHYGAERLGATVIPMSGGQTEKQA----QLIRDFQPDMIMVTPSYCLN  190

Query  217  -IATLARYLEANQSYYPAKLKSIMTSSESLSAEDRQVIETRFGCKVFDWYGLFERVAAIG  275
             I  L R L  + S     L+  +  +E  +   R+ IE R G    D YGL E V   G
Sbjct  191  LIEELERQLGGDASG--CSLRVGVFGAEPWTQAMRKEIERRLGITALDIYGLSE-VMGPG  247

Query  276  ---QC--EHGRYHLLSDYSHVELLAAGDG-------RYELVGTNFNNQYFPLIRYKTGDH  323
               +C        +  D+ + E++   DG         EL+ T    +  P+IRY+T D
Sbjct  248  VAMECLETTDGPTIWEDHFYPEIVNPHDGTPLADGEHGELLFTTLTKEALPVIRYRTRDL  307

Query  324  VHLSENEACPCGRVFPVIERIEGRIGDYLV  353
              L    A    R    ++RI GR  D L+
Sbjct  308  TRLLPGTA----RTMRRMDRISGRSDDMLI  333
```

RMSD between 154 pruned atom pairs is 1.281 angstroms; (across all 383 pairs: 9.276)

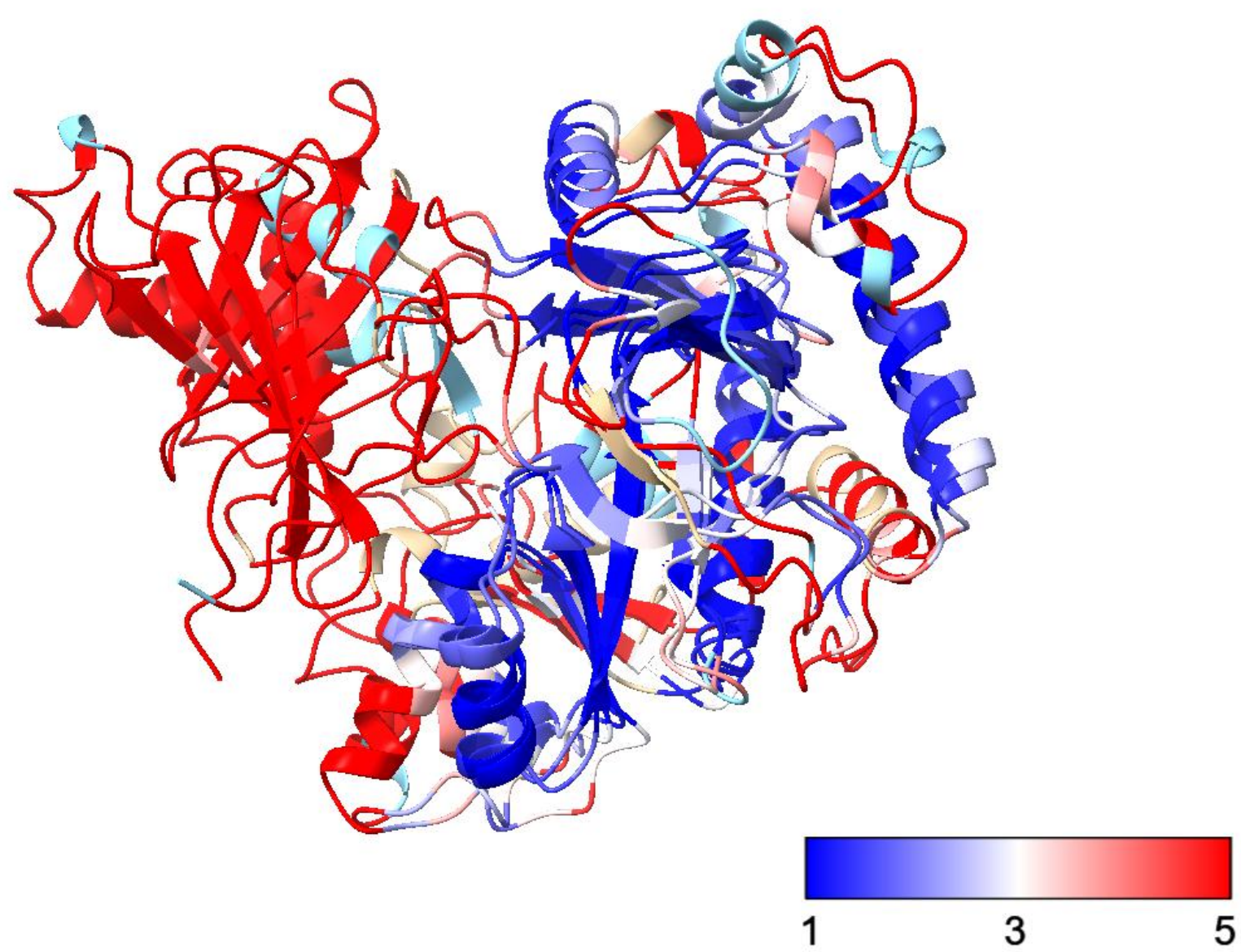

**Supporting Figure 7. Comparisons of acetyltransferases VpsC and VpsG with WcaB from *E. coli***

**VpsC (Uniprot ID: A0A2S1ZSY7)**

MSSPWELIKADLYRHTGQVSIGLVVKHLLLNAGFAYMFWFRLCRSKNVLIRTVARLMHRILSVRYQIQLPKETQVGPGLYLGHATGVIVNSTAKIGANCNLSPFTVIGSSQGQAATVGDCVYIGPHVSIVEDITIGDGSIIGAGSVVIRDVPPNSVVVGNPGRVLTRPSHKTYIRHPAPLESKS

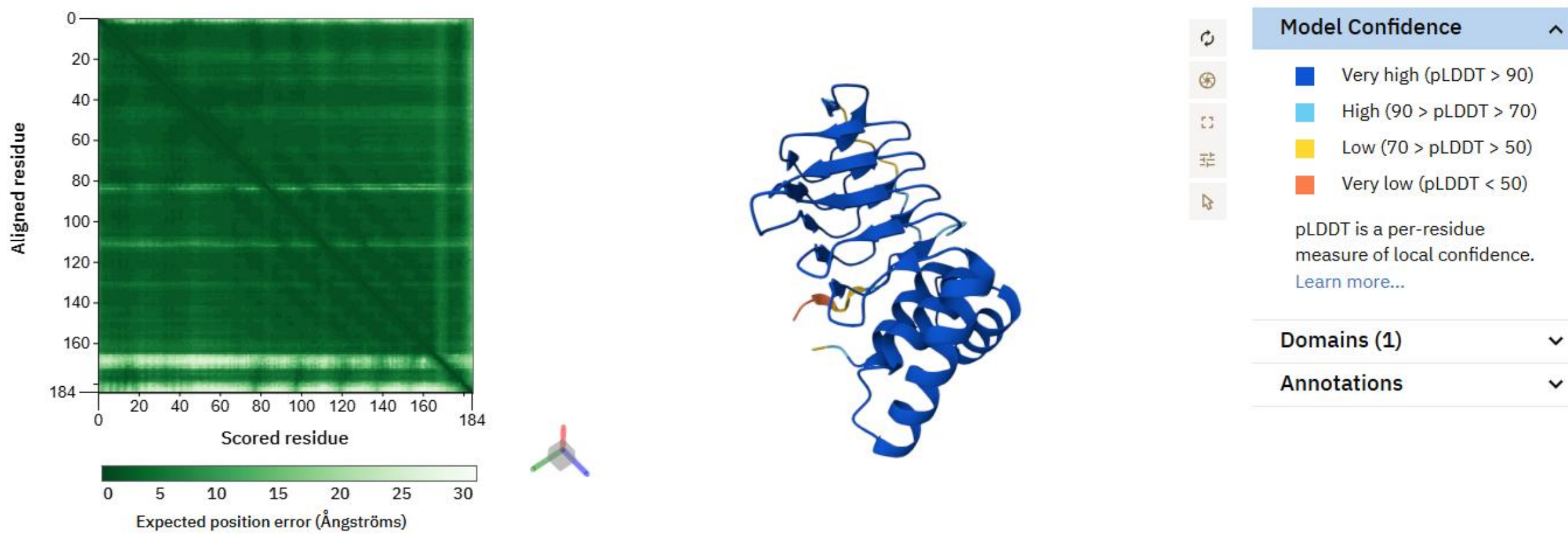


**VpsG (Uniprot ID: A0A086SS47)**

MWLYRVGHFFHKRRIPLLPQVCNGLIRLVHNCAVYSQSEIGKGTVFGYGGIAVVIHKRCVIGKECVIGSNVTIGGRSRSHNVPVIGNYVYIATGAKVLGDIRVGDGAVIGANAVVLEDVPPYSVVVGMPAKVIKTNINPKDFY

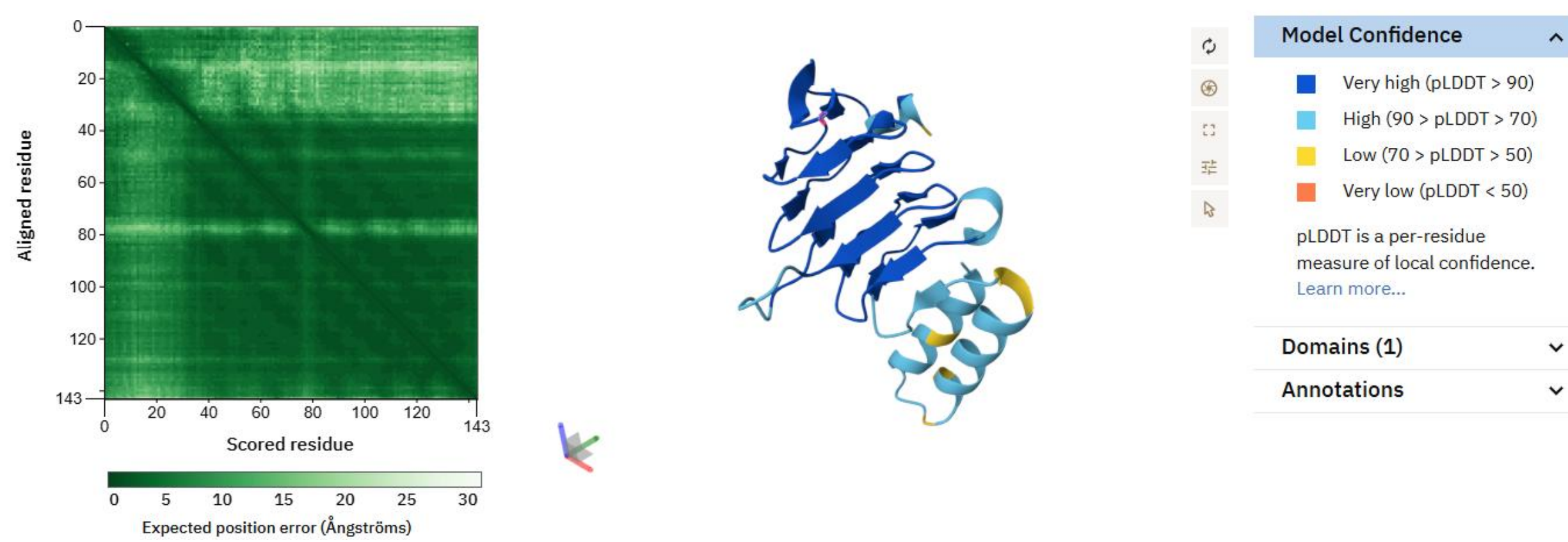


**WcaB *E. coli* (Uniprot ID: P0ACC9)**

MLEDLRANSWSLRPCCMVLAYRVAHFCSVWRKKNVLNNLWAAPLLVLYRIITECFFGYEIQAAATIGRRFTIHHGYAVVINKNVVAGDDFTIRHGVTIGNRGADNMACPHIGNGVELGANVIILGDITLGNNVTVGAGSVVLDSVPDNALVVGEKARVKVIK

**VpsC to WcaB**

| Score | Expect | Method | Identities | Positives | Gaps |
|---|---|---|---|---|---|
| 60.1 bits(144) | 5e-17 | Compositional matrix adjust. | 50/144(35%) | 74/144(51%) | 21/144(14%) |

```
Query  4    YRVGHF---FHKRRI-------PLLPQVCNGLIRLVHNC----AVYSQSEIGKGTVFGYG  49
            YRV HF   + K+ +       PLL      L R++  C     + + + IG+     +G
Sbjct  21   YRVAHFCSVWRKKNVLNNLWAAPLLV-----LYRIITECFFGYEIQAAATIGRRFTIHHG  75

Query  50   GIAVVIHKRCVIGKECVIGSNVTIGGR-SRSHNVPVIGNYVYIATGAKVLGDIRVGDGAV  108
              AVVI+K  V G +  I   VTIG R + +   P IGN V +     +LGDI +G+
Sbjct  76   -YAVVINKNVVAGDDFTIRHGVTIGNRGADNMACPHIGNGVELGANVIILGDITLGNNVT  134

Query  109  IGANAVVLEDVPPYSVVVGMPAKV  132
            +GA +VVL+ VP  ++VVG  A+V
Sbjct  135  VGAGSVVLDSVPDNALVVGEKARV  158
```

RMSD between 96 pruned atom pairs is 0.646 angstroms; (across all 155 pairs: 8.485)

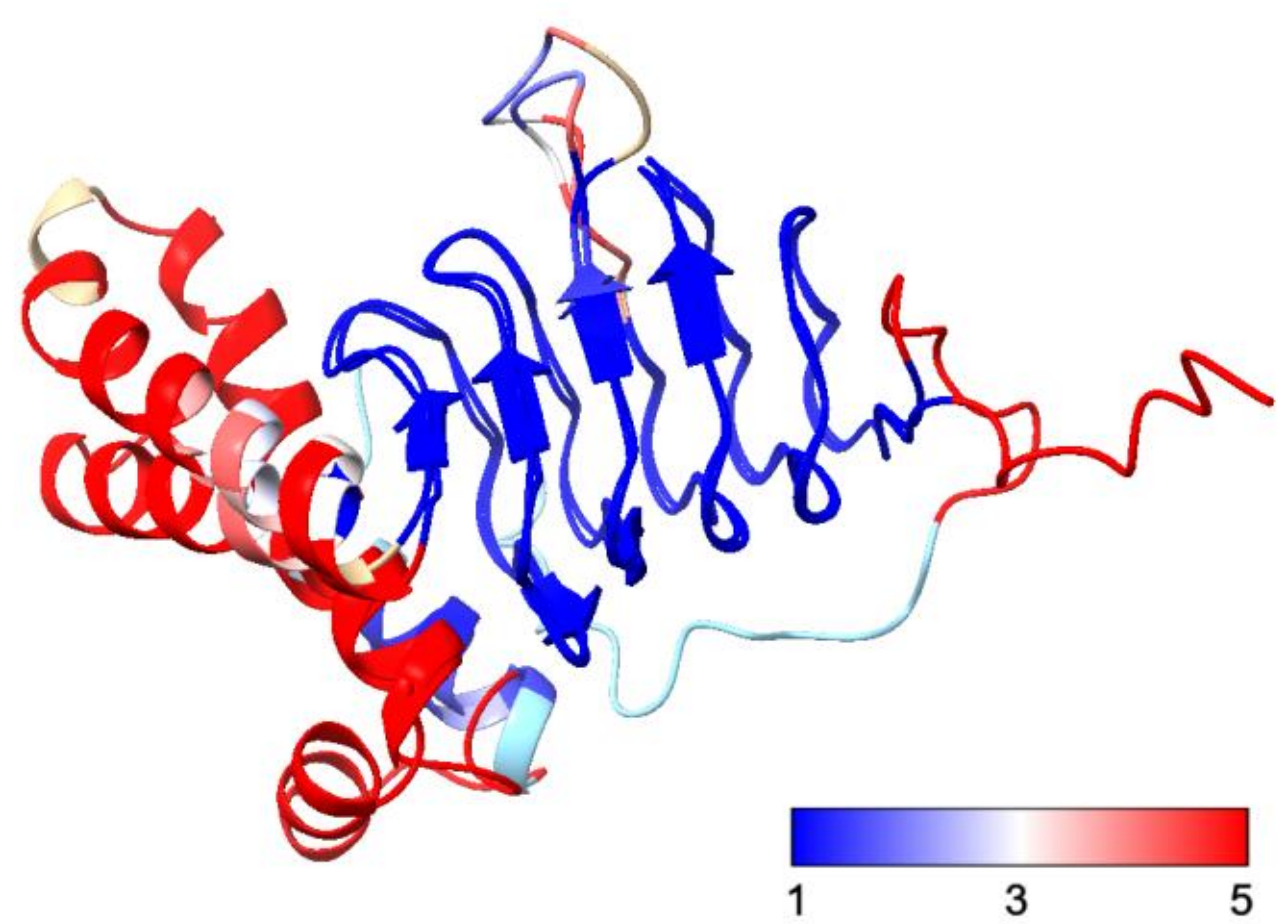

**VpsG to WcaB**

| Score | Expect | Method | Identities | Positives | Gaps |
| --- | --- | --- | --- | --- | --- |
| 53.5 bits(127) | 3e-14 | Compositional matrix adjust. | 41/130(32%) | 65/130(50%) | 11/130(8%) |

```
Query  44   RSKNVLIRTVAR---LMHRILS---VRYQIQLPKETQVGPGLYLGHATGVIVNSTAKIGA  97
            R KNVL    A    +++RI++     Y+IQ      +G    + H   V++N     G
Sbjct  31   RKKNVLNNLWAAPLLVLYRIITECFFGYEIQ--AAATIGRRFTIHHGYAVVINKNVVAGD  88

Query  98   NCNLSPFTVIGSSQGQAAT---VGDCVYIGPHVSIVEDITIGDGSIIGAGSVVIRDVPPN  154
            +  +     IG+          +G+ V +G +V I+ DIT+G+   +GAGSVV+  VP N
Sbjct  89   DFTIRHGVTIGNRGADNMACPHIGNGVELGANVIILGDITLGNNVTVGAGSVVLDSVPDN  148

Query  155  SVVVGNPGRV  164
            ++VVG   RV
Sbjct  149  ALVVGEKARV  158
```

RMSD between 97 pruned atom pairs is 0.739 angstroms; (across all 135 pairs: 7.550)

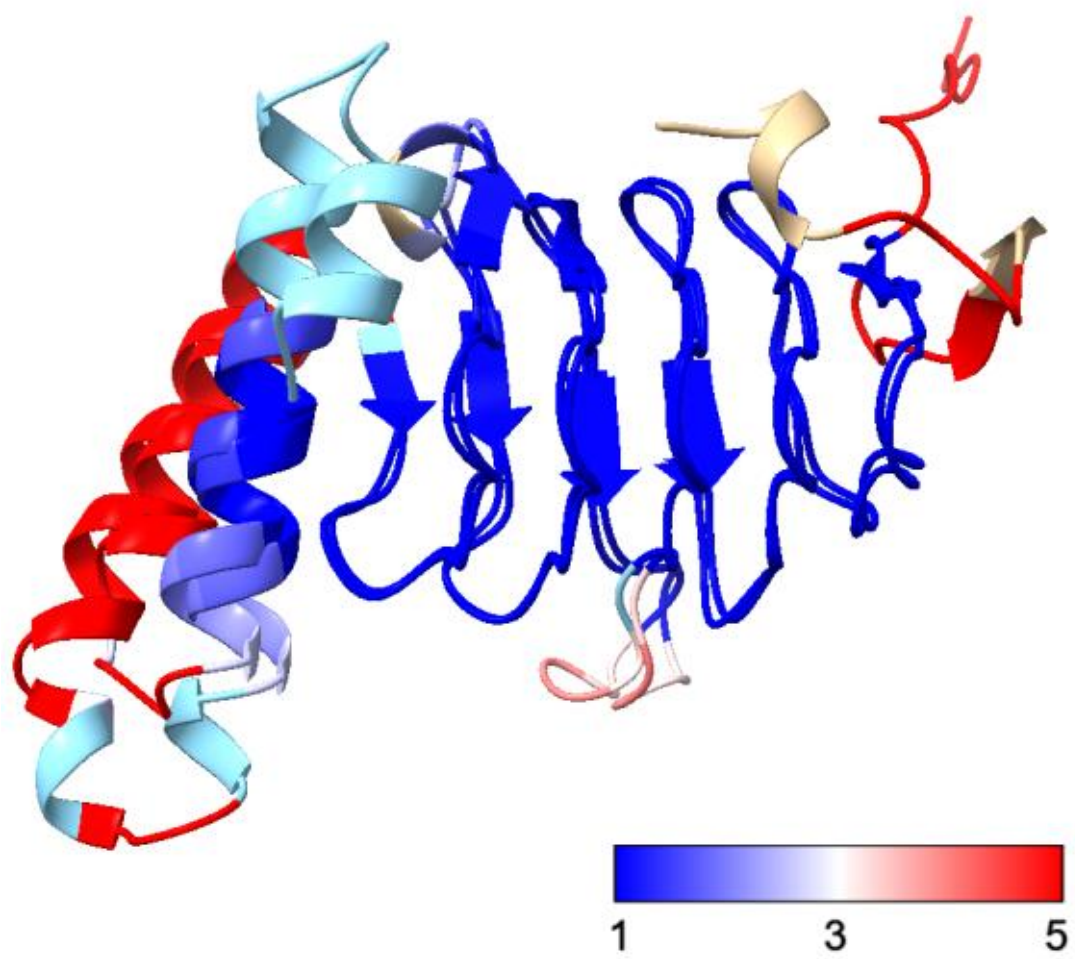

**Supporting Figure 8. Overlay of key residues of Cellobiose-2-epimerase (CE) and VpsJ.** Blue residues are VpsJ and Beige are CE. Labels use three letter code for VpsJ and one letter code for CE.

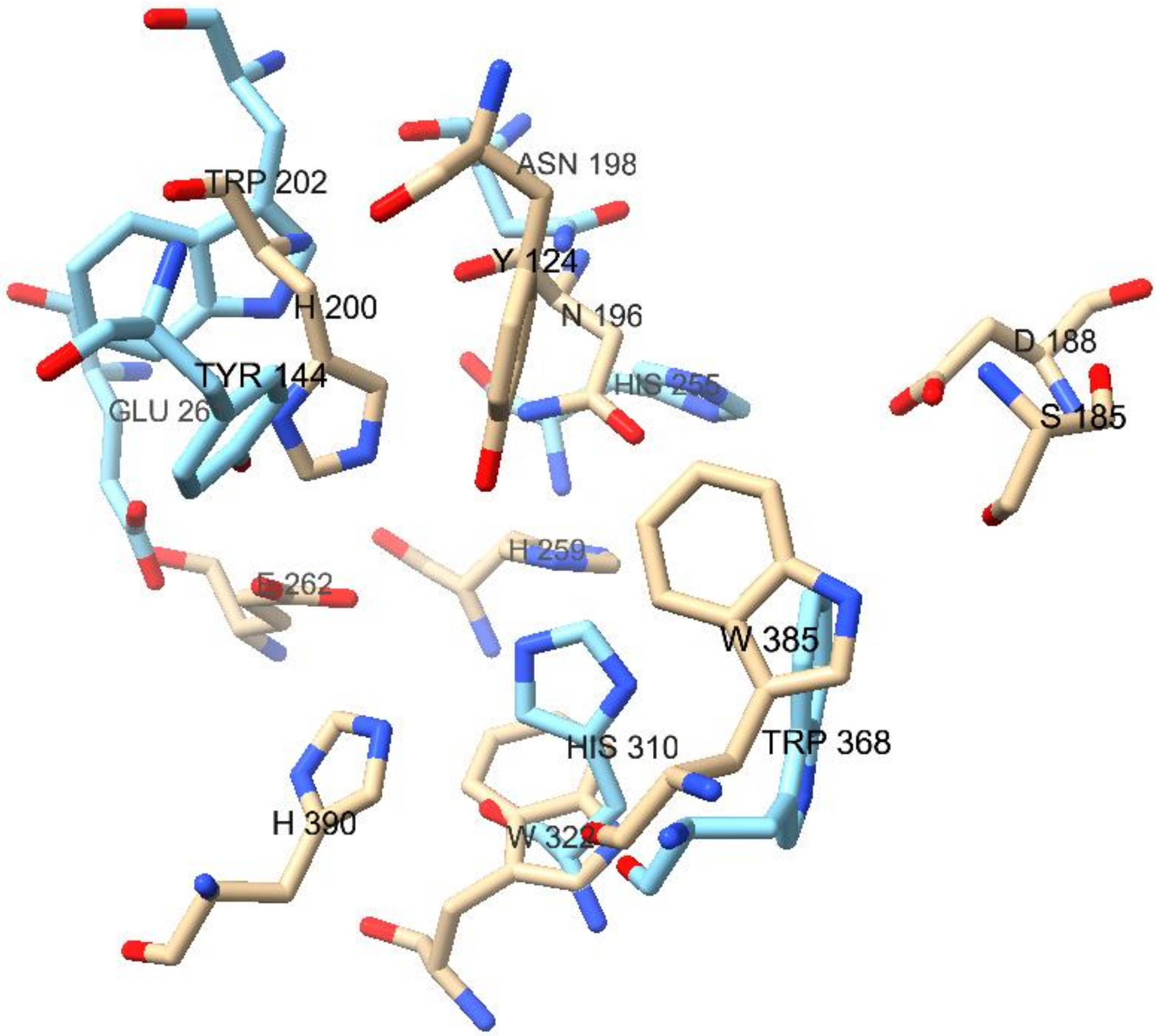

1. de Lorenzo V, Timmis KN. 1994. Analysis and construction of stable phenotypes in gram-negative bacteria with Tn5- and Tn10-derived minitransposons. Methods Enzymol 235:386-405.
2. Yildiz FH, Schoolnik GK. 1999. Vibrio cholerae O1 El Tor: identification of a gene cluster required for the rugose colony type, exopolysaccharide production, chlorine resistance, and biofilm formation. Proc Natl Acad Sci U S A 96:4028-33.
3. Beyhan S, Yildiz FH. 2007. Smooth to rugose phase variation in Vibrio cholerae can be mediated by a single nucleotide change that targets c-di-GMP signalling pathway. Mol Microbiol 63:995-1007.
4. Fong JCN, Syed KA, Klose KE, Yildiz FH. 2010. Role of Vibrio polysaccharide (vps) genes in VPS production, biofilm formation and Vibrio cholerae pathogenesis. Microbiology (Reading) 156:2757-2769.
5. Furste JP, Pansegrau W, Frank R, Blocker H, Scholz P, Bagdasarian M, Lanka E. 1986. Molecular cloning of the plasmid RP4 primase region in a multi-host-range tacP expression vector. Gene 48:119-31.
